# Microwave-Assisted Syntheses of Bioactive Seven-Membered, Macro-Sized Heterocycles and Their Fused Derivatives

**DOI:** 10.3390/molecules21081032

**Published:** 2016-08-09

**Authors:** Mohsine Driowya, Aziza Saber, Hamid Marzag, Luc Demange, Khalid Bougrin, Rachid Benhida

**Affiliations:** 1 Laboratoire de Chimie des Plantes et de Synthèse Organique et Bioorganique, URAC23, Faculté des Sciences, Université Mohammed V, B.P. 1014 Rabat, Maroc; m.driowya@hotmail.com (M.D.); aziza.saber@ymail.com (A.S.); marzaghamid@gmail.com (H.M.); 2 Institut de Chimie de Nice, ICN UMR UNS CNRS 7272, Université Nice-Sophia Antipolis-Université Côte d’Azur, Parc Valrose, 06108 Nice Cedex 2, France; 3 Département de Chimie, Université Paris Descartes, Sorbonne Paris Cité, UFR des Sciences Pharmaceutiques, 4 avenue de l’Observatoire & UFR Biomédicale des Saints Pères, 45 Rue des Saints Pères, Paris Fr-75006, France

**Keywords:** microwave irradiation, bioactive molecules, dipolar cycloaddition, cyclocondensations, multicomponent reactions

## Abstract

This review describes the recent advances in the microwave-assisted synthesis of 7-membered and larger heterocyclic compounds. Several types of reaction for the cyclization step are discussed: Ring Closing Metathesis (RCM), Heck and Sonogashira reactions, Suzuki-Miyaura cross-coupling, dipolar cycloadditions, multi-component reactions (Ugi, Passerini), etc. Green syntheses and solvent-free procedures have been introduced whenever possible. The syntheses discussed herein have been selected to illustrate the huge potential of microwave in the synthesis of highly functionalized molecules with potential therapeutic applications, in high yields, enhanced reaction rates and increased chemoselectivity, compared to conventional methods. More than 100 references from the recent literature are listed in this review.

## 1. Introduction

During the last decade, the continuous advances in microwave (MW) assisted syntheses have highlighted the huge potential of this technique for large-scale preparations of natural products and therapeutic agents. MW processes are highly compatible with the main reactions used in modern organic chemistry, such as ring closure metathesis (RCM), multi-component reaction (MCR), metal catalysed reaction (Heck, Ullmann, Sonogashira, Suzuki-Miyaura, etc), cyclocondensations, dipolar cycloadditions, etc. Moreover, MW technology has also been widely used for the development of eco-friendly and solvent-free syntheses of multi-functionalized molecules [1,2]. Indeed, more than 250 articles are devoted yearly to MW-assisted syntheses, an approach whose popularity is related not only to its low costs and its experimental simplicity but also to its compatibility with very mild conditions to perform sensitive reactions. Moreover, MW procedures are often associated with improved yields; enhanced chemoselectivity and purity compared to conventional procedures.

These benefits are mainly due to the physical characteristics of MW irradiation, which allow not only a better transmission of the energy to the reactants but also an homogeneous heating of the solution mixtures. In addition, the electric fields generated by the MWs cause oscillations and rotations of dipolar and ionic molecules, resulting in an additional local heating. This specific dielectric heating of polar reactants, so-called “specific microwave” or “non-thermal” effect, is often considered when MW irradiation leads to different products from the ones obtained through conventional heating at the same apparent temperature. 

We recently reviewed the MW-assisted synthesis of 4-, 5- and 6-membered heterocycles [3,4]. The specific purpose of this review, mainly focused on results published during the last decade, is to highlight the MW applications to macrocyclic heterocycles. To this end, the first part of this review will be devoted to the synthesis of 7-membered heterocycles containing one, two or three heteroatoms (nitrogen, oxygen and sulphur). In the second part, selected syntheses of macrocycles (peptides, pseudo-peptides and calix-type molecules) will be discussed.

## 2. Synthesis of Heterocyclic Compounds

### 2.1. Seven-Membered Heterocycles

#### 2.1.1. Seven-Membered Heterocycles with One Heteroatom

##### Benzazepines and Fused Analogues

**Figure 1:**
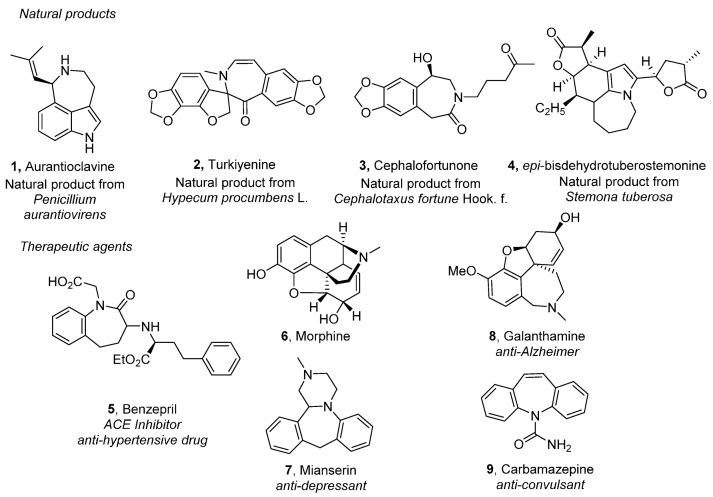
Representative examples of bioactive benzazepines.

The benzazepines form a class of seven-membered aza-heterocycles fused with an aromatic ring, which is divided into the three distinct isomeric forms (1-, 2- and 3-benzazepines), depending on the relative position of the nitrogen atom. A huge variety of bioactive molecules, including natural products extracted from plants or fungus, contain a benzazepine core, as illustrated by the scaffold of morphine (a 3-benzazepine). Numerous series of benzazepine derivatives have been marketed for their therapeutic use as anti-depressant, anti-hypertensive, anti-ischaemic, and their pharmacological properties have been recently reviewed by various researchers [5,6,7,8,9] (Figure 1).

###### 2-Benzazepines and Their Derivatives

**Scheme 1:**
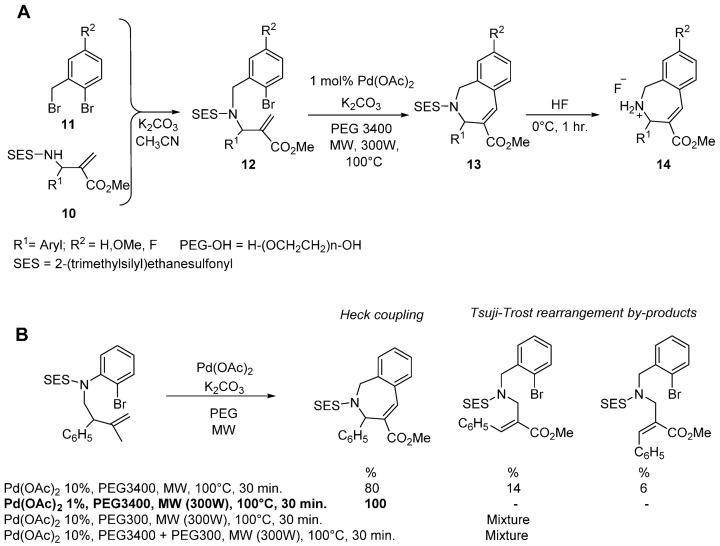
Heck reaction leading to 2-benzazepines.

Lamaty and co-workers have disclosed a palladium-catalysed Heck reaction under MW-heating in polyethyleneglycol (PEG) for the preparation of novel series of 2-benzazepines **14** starting from the protected β-aminoesters **12** (Scheme 1A) [10]. PEGs are low-toxicity solvents sharing common characteristics with ionic liquids, such as high polarity and high boiling points. Moreover, this synthesis is among the first reported procedures using PEGs in a MW-assisted reaction.

Interestingly, the profile of the temperature ramp exerts a strong influence on the chemoselectivity of this reaction. Indeed, two by-products resulting from a Tsuji-Trost rearrangement reaction have been observed (Scheme 1B). The highest rate of formation for these by-products occurs when the synthesizer's heating power at starting is not set to its maximum (300 W). In this case, several minutes are required to reach the appropriate temperature for the reaction. Moreover, the heating is delayed due to the time needed for the melting of PEG3400 (58–65 °C). This induces an inhomogeneity of the heating, leading to difficulties in reproducing the experiments. On the other hand, the use of low molecular weight PEGs (PEG300), which is not solid at room temperature, or the addition of DMF, leads to complicated mixtures of products. 

Finally, the authors demonstrated that using PEG3400 as solvent in presence of K_2_CO_3_ as base and 1% of PdOAc_2_ as catalyst, under MW-irradiation (300 W, 100 °C), results in the formation of the expected 2-benzazepine in 30 min, without formation of by-products. The conversion rates are good (up to 80%), and the work-up conditions are simplified (precipitation of the PEG/Pd/base through addition of diethyl ether). Moreover, the catalytic system can be recycled, even if the conversion rates are reduced during the second reaction cycle.

**Figure 2:**
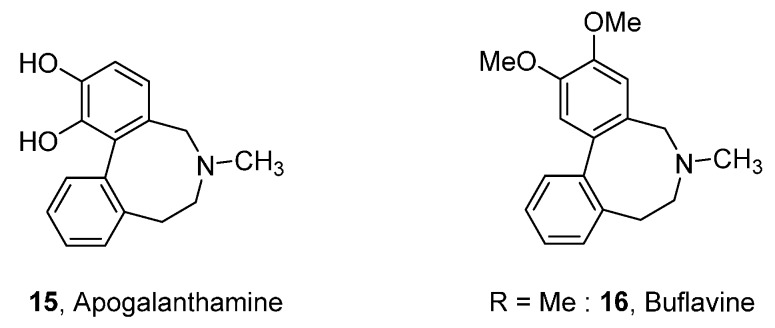
Representative examples of bioactive 5,6,7,8-tetrahydrodibenzo[*c*,*e*]azocine.

The 6,7-dihydro-5*H*-dibenzo[*c*,*e*]azepines are structurally-related to the natural 5,6,7,8-tetrahydrodibenzo[*c*,*e*]azocine skeleton, an unusual motif which consists on a biarylic ring system linked through a 8 or 7 membered *N*-heterocycle. This core has been described in the structure of few natural products, whose leading members are (i) apogalanthamine (**15**), an alkaloid from the Amaryllidaceae family and (ii) buflavine (**16**), a natural extract from *Boophane flava* (Figure 2) [11,12].

**Scheme 2:**
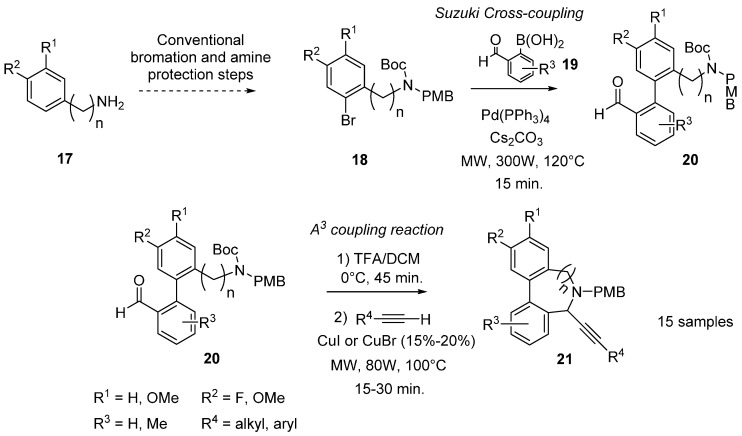
Suzuki-Miyaura reaction/A^3^ coupling sequence leading to 2- and 3-benzazepines.

An interesting synthesis of this scaffold has been reported by Van der Eycken’s group at Leuven. The two key steps of this synthesis are (i) a Suzuki-Miyaura cross-coupling reaction; and (ii) an A^3^ intramolecular coupling reaction, each of which occurs under MW-activation (Scheme 2) [13,14]. Remarkably, despite of the electronically unfavourable combination of the *o*-bromobenzylamine **18** and the electron-poor *o*-formylarylboronic acid **19**, the MW-activation of the Suzuki coupling leads to the biaryl **20** (95% yield) in only 15 min. In the next step, the coupling between the secondary amine, the aldehyde and the acetylenic derivatives (so-called A^3^ coupling), occurs under MW-irradiation using Cu(I) (20% max.) as catalyst, and affords the required heterocyclic compounds **21** in 20–30 min and in high yields (up to 90%). For this latter step, CuI, CuBr and CuCl can be used as Cu^I^ salts; interestingly, a decrease of the catalyst amount results in increased reaction times, but not in decreased conversion rates. 

###### 3-Benzazepines and Their Derivatives

**Figure 3:**
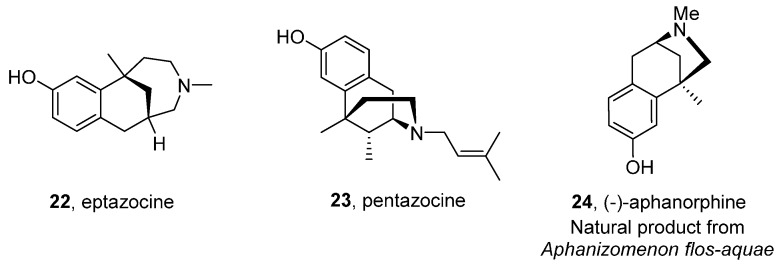
Representative examples of bioactive 3-benzazepines.

The use of MW-activation for the synthesis of 3-benzazepines and derivatives has been extensively studied by Van der Eycken, as illustrated by an elegant asymmetric approach towards the alkaloid (−)-aphanorphine (**24**), which is structurally-related to two analgesics: pentazocine (**23**) and eptazocine (**22**) (Figure 3).

**Scheme 3:**
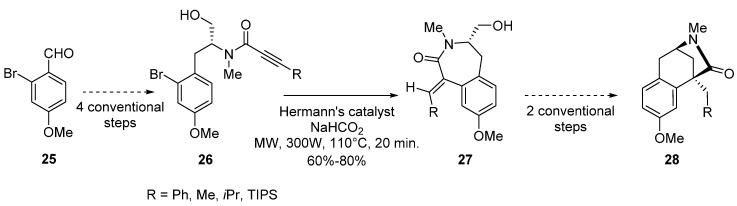
Synthesis of 3-benzazepines through Heck intramolecular reaction.

In this synthesis, the MW-assisted Heck reaction allowed the cyclisation of the 3-benzazepine ring **27**, as outlined in Scheme 3 [15]. This intramolecular coupling has been carried out by applying a maximum power level of 300 W at 100 °C for 20 min, in presence of Hermann’s palladacycle as catalyst (2.5%) and NaHCO_2_ as reducing agent.

The expected benzazepines have been isolated in good yields (60%–80%). Nevertheless, when the alkyne is substituted by a bulky group (**26**, R = TIPS), these MW conditions gave benzazepine **27** in only 7% yield. This is probably due to the reduction of the bromine, and its subsequent elimination. For this specific case, the cyclisation required the use of standard conditions (65 °C, conventional heating for 5 h) to reach 70% yield.

**Scheme 4:**
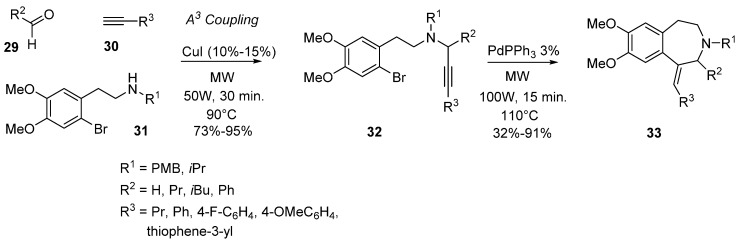
A^3^ coupling/Pd-catalyzed cyclization sequence leading to 3-benzazepines.

In the same group, Peshkov and co-workers have reported a fully-stereoselective two-step synthesis of 3-benzazepines, through an A^3^ multi-component coupling/intramolecular cyclisation sequence; each step of this sequence occurs under MW irradiation. Indeed, the first A^3^-coupling consists on mixing the aldehyde **29**, the alkyne **30** and the amine **31** in presence of Cu(I) catalysis (CuI or CuBr, 15%) and under MW-irradiation (90 °C, 50 W, 30 min). This reaction affords the functionalized propargylamines **32** in high yields (73%–95%). Importantly, this A^3^ coupling doesn’t occur under conventional heating. In the second step, the propargylamines **32** undergo a stereoselective Pd-catalysed (PdPPh_3_, 3%) intramolecular hydroarylation reaction of the acetylene group under MW-irradiation (110 °C, 100 W, 15 min), leading to the expected 3-benzazepines in 32-91% yield (**33**, Scheme 4) [16]. 

**Scheme 5:**
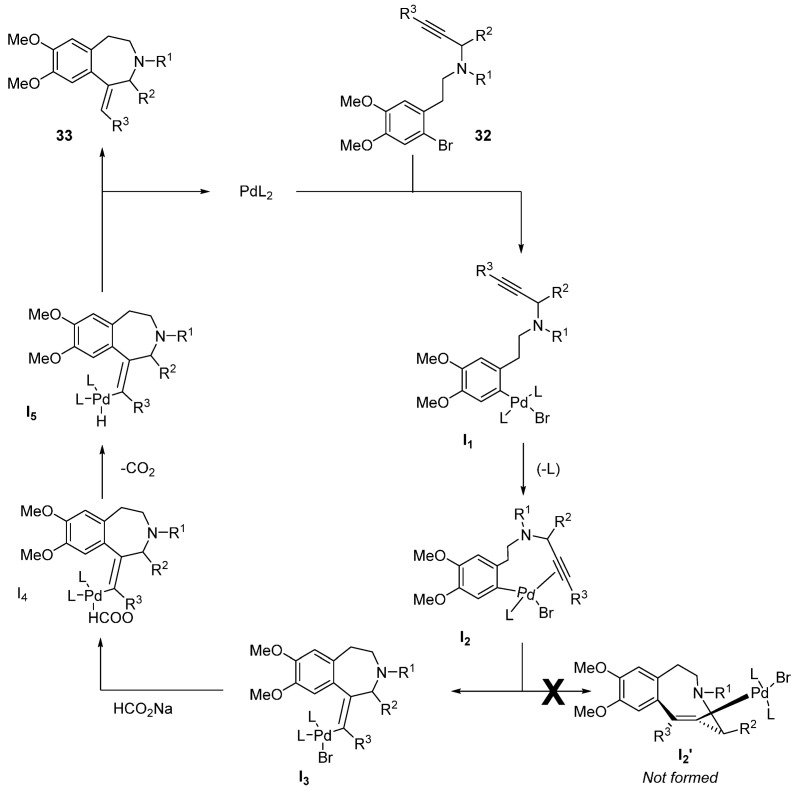
Mechanistic study of the 3-benzazepines synthesis reported by Peshkov and co-workers [16].

The mechanism of the cyclisation step is outlined in Scheme 5. In this hypothetic pathway, the arylpalladium π-complex **I_2_** undergoes a *syn*-addition to the triple bond leading to the σ-vinyl palladium complex **I_3_**. The authors hypothesized that the high strain exerted by the trans-geometry around the double bond prevents the *endo*-cyclisation through the potential complex **I_2_’**. Therefore, this synthesis is fully stereoselective, leading only to an exocyclic bond with a *Z*-configuration.

An alternative route to 3-benzazepines consists on the use of 3-benzazepin-2-ones as key intermediates. In a study reported by Sarkat and co-workers [17], the condensation of aromatic keto acids **34** with primary amines afforded the 3-benzazepin-2-ones **35**. The use of conventional heating (refluxing toluene), large excess of amine (up to 10 eq.) and/or long reaction times (more than 3 days) yields the required products in only 20% in the best cases. By a marked contrast, MW-activation (150 W, 5 bars) leads to the heterocycles in only 3 h with improved conversion rates (30%–60% yield). Importantly, the formation of the 3-benzazepinone does not occur when using a primary amine bearing two substituents in the α-position. For example, when using (*S*)-1-phenylethylamine, the authors reported the formation of the indalone **38** instead of the expected seven-membered heterocycle. In this case, the authors hypothesized that the double bond of the intermediate enamine **37** reacted faster with the carboxyl group than the amino moiety.

**Scheme 6:**
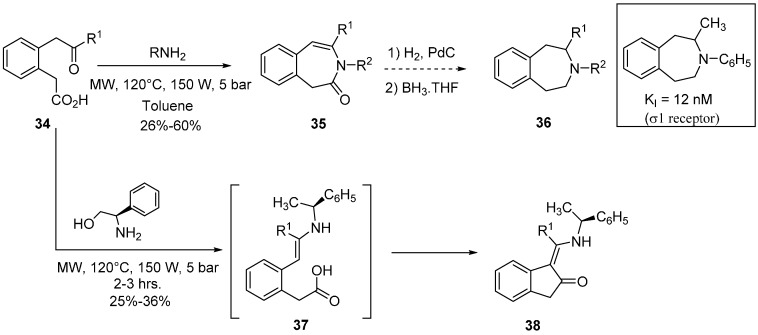
MW-assisted synthesis of 3-benzazepines and indalones with a potential activity against CNS disorders.

Lastly, the hydrogenation of the 3-benzazepinones **35** in presence of Pd/C followed by the reduction of the lactam, using BH_3·_THF complex, leads to racemic 2,3-disubstituted tetrahydro-3-benzazepines **36** in good yields. These molecules have been studied as ligands for the σ1 receptor, which is distributed in the central nervous system as well as tissues like kidney, liver, lung and heart. The leading compound binds this receptor with a K_i_ = 12 nM, underlying the potencies of the 3-benzazepine scaffold for the design of new therapeutic agents directed toward CNS disorders (Scheme 6) [17].

##### Oxepanes, Thiepanes and Their Derivatives

**Figure 4:**
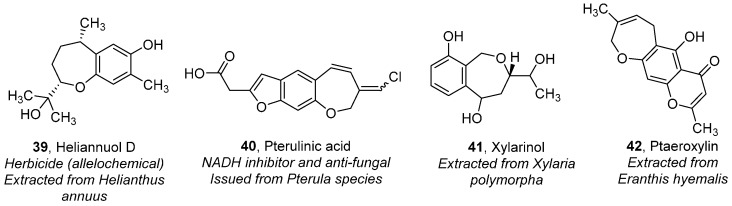
Relevant examples of bioactive benz[*b*]oxepines.

The benz[*b*]oxepines form a class of seven-membered heterocycles, containing one oxygen atom, which are fused to an aromatic moiety. This scaffold arises only in a small set of bioactive natural products produced by plants (Figure 4) [18,19,20,21].

**Scheme 7:**
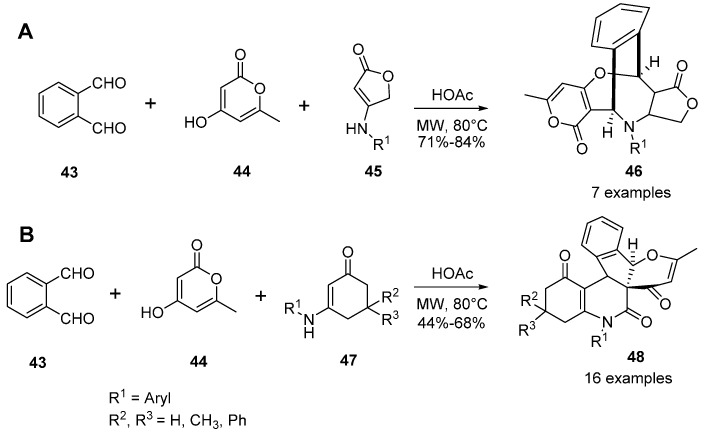
One-pot, multi-component reaction leading to pentacyclic pyrano[4,3-*b*]oxepines.

**Scheme 8:**
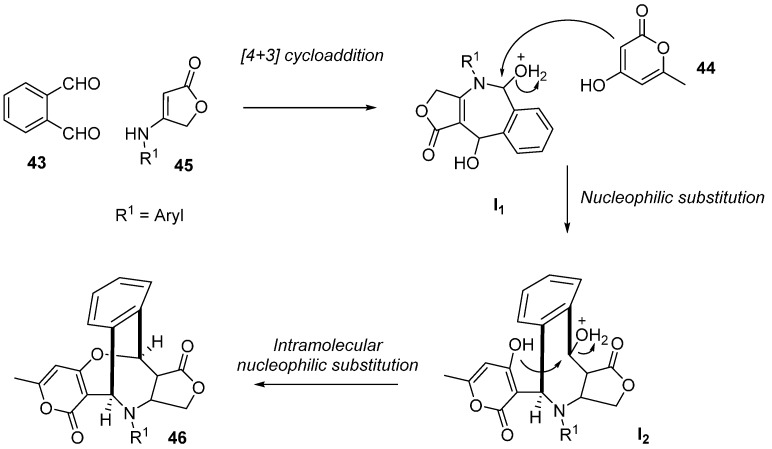
Mechanistic study of the benz[*b*]oxepines synthesis reported by Jiang and co-workers [22].

To the best of our knowledge, very few syntheses of benz[*b*]oxepines have been reported. In this context, the efficient domino-strategy elaborated by Jiang and co-workers leading to series of ([3,4]furanimino)benzo[*e*]pyrano[4,3-*b*]oxepines is a remarkable exception [22]. In this approach, the one-pot, MW-assisted, multi-component reaction involving *o*-phtalaldehyde **43**, 4-hydroxy-6-methyl-2*H*-pyranone **44** and *N*-substituted 4-aminofuran-2(5*H*)-ones **45** leads to a series of pentacyclic pyrano[4,3-*b*]oxepines **46** (Scheme 7A) in high yields (up to 70%) and short reaction times (less than 30 min). According to the authors’ hypothesis, the reaction occurs through a first [4 + 3] cycloaddition between the *o*-phtalaldehyde **43** and the aminofuranone **45** leading to the azapinediol **I_1_** which undergoes next a double intramolecular nucleophilic substitution leading to the pentacyclic pyrano[4,3-*b*]oxepines **46.** This reaction occurs with remarkable chemo-, stereo- and regioselectivities (Scheme 8). It is to note that, by a similar way, a series of multi-functionalized pyrano[3′,2′,2,3]indeno[2,1]quinolones **48** (Scheme 7B) has been obtained using six-membered cyclic enaminones **47** instead of the aminofuranone **45**.

**Scheme 9:**
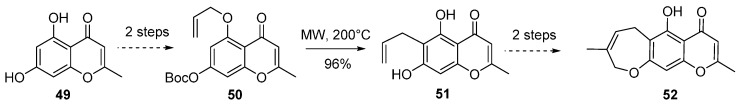
Synthetic route to ptaeroxilin involving a MW-assisted Claisen rearrangement step.

Another interesting route to benzoxepines has been proposed by Moody’s group, which reported the synthesis of the ptaeroxilin **52** and its derivatives. These molecules belong to the family of the natural oxepinochromones. However, these authors do not take advantage of the MW-irradiation for the closure of the seven-membered heterocycle, but they used a MW-assisted Claisen re-arrangement, which occurs concomitantly with a Boc deprotection leading to the key intermediate **51** (Scheme 9) [23].

**Figure 5:**
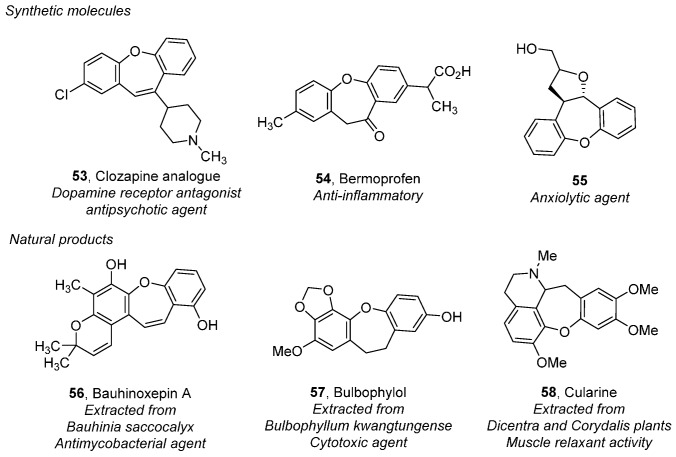
Relevant examples of bioactive dibenz[*b*,*f*]oxepines.

The dibenz[*b*,*f*]oxepines are characterized by an oxepane fused with two aromatic moieties: this is a relevant pharmacophore present in the structure of numerous therapeutic agents which can be used for several therapeutic applications such as antidepressant, antipsychotic and anti-inflammatory indications (Figure 5) [24,25,26,27,28].

**Scheme 10:**
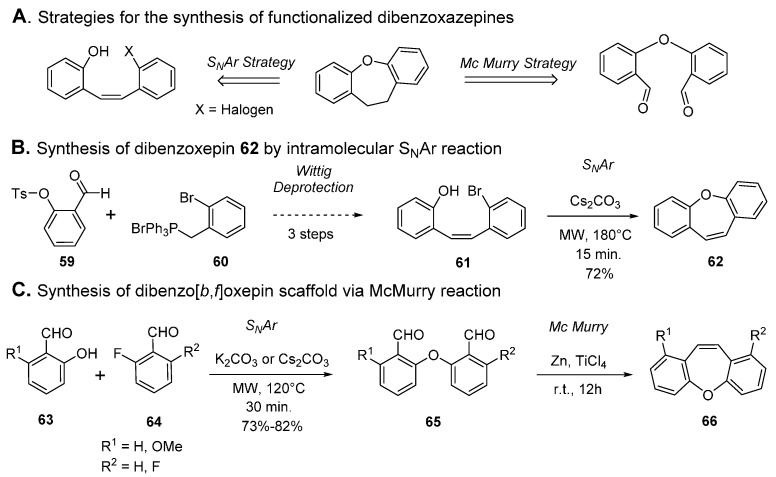
Two MW-assisted synthesis of functionalized dibenzoxazepines.

Very few MW-assisted syntheses of dibenzoxazepines have been reported. Nevertheless, two very interesting approaches, based (i) on an intramolecular S_N_Ar; and (ii) on a McMurry reaction, each occurring with MW-activation, have been described by Moreno and co-workers [29] (Scheme 10A).

The S_N_Ar strategy requires the synthesis of a multi-functionalized *Z*-stilbene **61**, which is obtained in 87% yield through a conventional Wittig reaction involving *o*-bromobenzyl-triphenylphosphonium salt **60** and 2-formylphenyl-4-methylbenzene sulfonate **59**. Subsequent cleavage of the *p*-toluenesulfonate group leads to a *Z*-stilbene **61**, which undergoes an intramolecular S_N_Ar reaction under MW-irradiation. The resulting dibenzo[*b*,*f*]oxepin **62** is obtained in 72% yield (Scheme 10B).

The alternative strategy consists of the intramolecular McMurry condensation of aromatic diaryl ethers **65**. These intermediates are synthesized in high yields (up to 75%) through MW-assisted S_N_Ar reactions, carried out at 120 °C in DMSO or DMA and in the presence of K_2_CO_3_ or Cs_2_CO_3_ as base (2.0 eq.) (Scheme 10C). It is important to note that, in this case, the S_N_Ar reaction could also be performed under conventional heating but under harsher conditions that generally lead to low conversion rates and poor yields.

**Figure 6:**
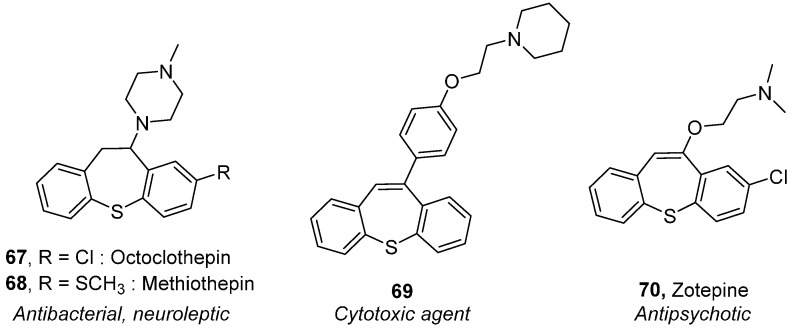
Relevant examples of bioactive dibenzo[*b*,*f*]thiepines.

The dibenzo[*b*,*f*]thiepines are a pharmacologically relevant family of seven-membered heterocycles containing one sulfur atom. Indeed, several examples of dibenzo[*b*,*f*]thiepines with anti-bacterial, anti-psychotic, neuroleptic or anti-proliferative activities have been highlighted in the literature as illustrated in Figure 6 [30,31,32].

**Scheme 11:**
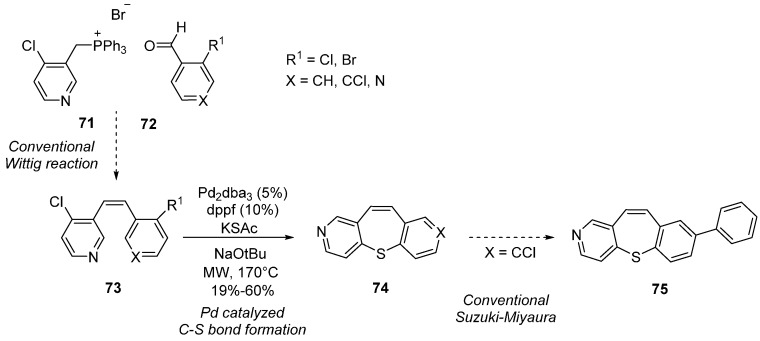
Synthesis of dibenzo[*b*,*f*]thiepines by means of a Pd-catalyzed double C-S bond formation.

Nevertheless, at the best of our knowledge, only one example of MW-assisted synthesis of a dibenzo[*b*,*f*]thiepine has been reported in the literature. The originality of this strategy, disclosed by Bozinovic and co-workers [33,34], consists in the use of potassium thioacetate (KSAc) as a sulfur source for a double palladium-catalyzed C-S bond formation (Scheme 11). Indeed, the use of this reactant on functionalized stilbenes **73** (accessible through conventional Wittig reactions) in presence of a suitable mixture of Pd(0) catalyst and ligand, affords the required dibenzo[*b*,*f*]thiepines **74**. The use of MW-activation for this double C-S bond formation reduces the reaction times (from 14 h to 90 min) and improves the conversion rates (from 30% to 50% approx.). Interestingly, this coupling is compatible with different sources of Pd(0), including Pd(OAc)_2_ and Pd_2_dba_3_, but is less versatile regarding the nature of the ligand. For example, no reaction occurs in presence of JohnPhos, a ligand that has been successfully used for a similar palladium-catalyzed double amination-cyclization reaction leading to corresponding azepines derivatives. In this context, the authors point out the relevance of 1,1′-bis(diphenylphosphino)ferrocene (dppf) as the suitable ligand for this double C-S bond formation.

In the next steps, the dibenzo[*b*,*f*]thiepines have been converted into the biphenyl derivatives **75** through conventional Suzuki-Miyaura cross coupling reactions. Among the final compounds, several molecules exhibited significant anti-fungal activities, in particular against *S. cerevisiae* and *A. brasiliensis*.

#### 2.1.2. Seven-Membered Heterocycles with Two Heteroatoms

##### Seven-Membered Heterocycles with Two Nitrogen atoms

**Figure 7:**
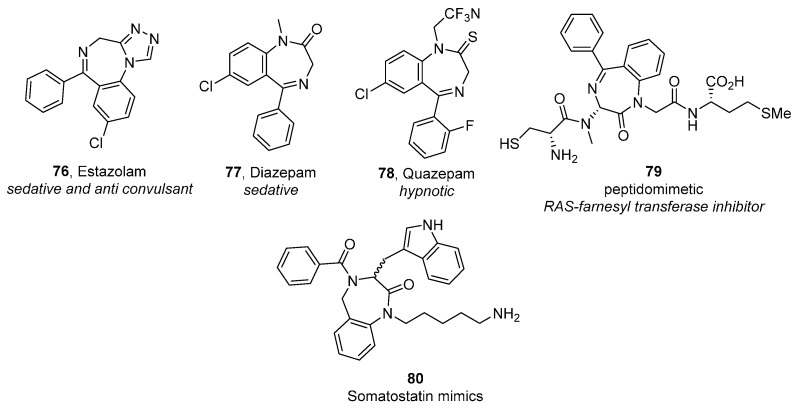
Relevant examples of bioactive benzodiazepines.

The diazepines and benzodiazepines form a class of seven-membered heterocycles, containing two nitrogen atoms, and fused with a benzenic core. Depending of the relative positions of the nitrogen atoms, several classes of diazepines and benzodiazepines are described. The benzodiazepines have been frequently used in drug design as β-turn or α-helix mimetics, and these motives are also used to constrain peptidic sequences. Therefore, this scaffold is considered as a privileged pharmacophore (Figure 7) [35,36,37].

In this context, interesting benzodiazepines preparations have been reported. However, the huge majority of these conventional syntheses are characterized by long reaction times, expensive reagents, harsh conditions, low-product yields, occurrence of several side products and difficulty to recover and/or re-use the catalysts. We report in this section new relevant examples of diazepines and benzodiazepines MW-accelerated synthesis, and their subsequent conversion and functionalization.

###### Diazepines

**Scheme 12:**
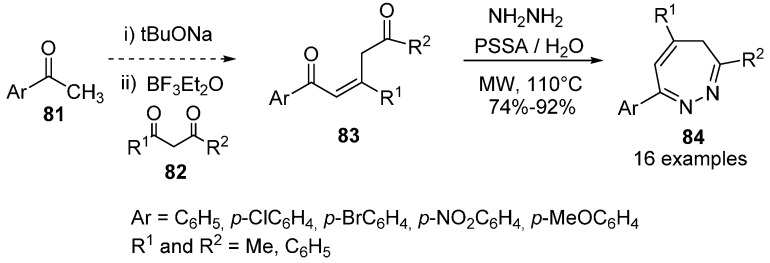
Intramolecular condensation of diketones in water and under polystyrene sulfonic acid leading to 1,2-diazepines.

A simple MW-assisted synthesis of the 1,2-diazepines **84** based on the condensation of α,β-unsaturated 1,5-diketones **83** with hydrazine has been reported by Manih et al. [38]. This reaction occurs at 110 °C in water and in the presence of polystyrene sulfonic acid (PSSA) as catalyst. The required products are isolated with high yields (up to 85%) and in short reaction time (less than 10 min). In this synthesis, the diketones have been obtained through the conventional condensation of acetophenones **81** with 1,3-diketones **82** (Scheme 12).

**Scheme 13:**
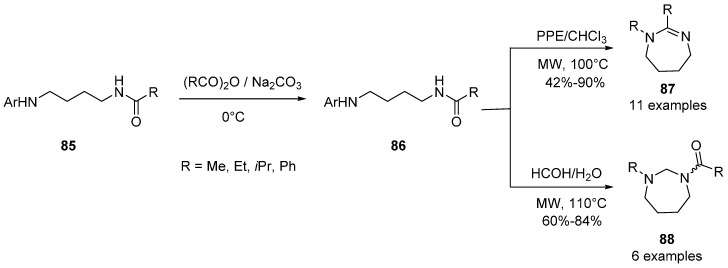
Synthetic route to 1,3-diazepines via cyclo-dehydration of aminoamides.

Orelli and co-workers [39,40] have developed a synthetic route to a series of 1,3-diazepines starting from the *N*-arylputrescine derivative **85**, which is quantitatively converted into the key aminoamides **86** by means of conventional acylation reactions. In marked contrast to conventional cyclodehydration reactions leading to seven-membered amidines, which require long reaction times and drastic heating, the MW-assisted cyclodehydration of **86** using a CHCl_3_ solution of ethyl-polyphosphate (PPE), a mild “Lewis acid” irreversible dehydrating reagent, leads to corresponding 1-aryl-2-alkyl-1*H*-1,4,5,6-tetrahydro-1,3-diazepines **87** in 8 min at 100 °C with good yields (42%–90%) (Scheme 13).

Otherwise, the MW-assisted cyclo-dehydration of **86** with aqueous formaldehyde in methanol afforded *N*-acyl-*N*′-arylhexahydro-1,3-diazepines **88** in only 2 min at 110 °C (Scheme 13).

**Scheme 14:**
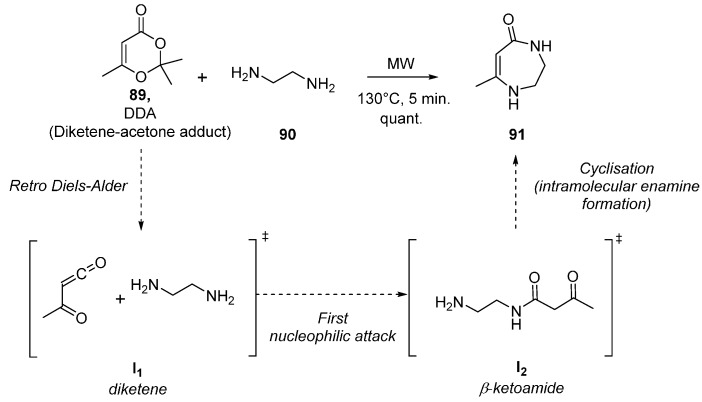
Solvent-free synthesis of 1,4-diazepines through Retro Diels-Alder and intramolecular enamine formation.

A solvent-free synthesis of 1,4-diazepines has been recently reported by Petros and co-workers [41]. In this preparation, 2,2,6-trimethyl-4*H*-1,3-dioxin-4-one **89** (the so-called diketene acetone adduct, DDA) and linear bisamines **90** were condensed at 130 °C under MW-irradiation in solvent-free conditions (Scheme 14). 

The corresponding diazepines **91** are obtained in only five minutes. Acetone and water are the sole by-products and are easily removed from the reaction mixture. Mechanistically, the reaction begins with a retro-Diels-Alder reaction leading to the diketene **I_1_**, which undergoes a nucleophilic attack by a linear bisamine to generate the intermediate β-keto amide **I_2_**. This intermediate **I_2_** undergoes an intramolecular cyclization through enamine formation (Scheme 14).

###### Diazepines Fused with Five-Membered Cycles

**Scheme 15:**
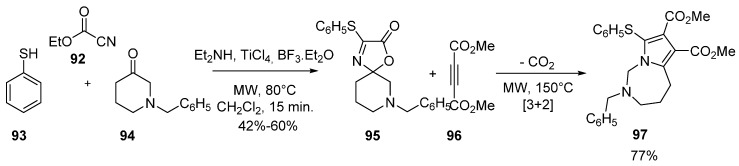
One-pot, three components reaction leading to pyrrole-fused 1,3-diazepines.

An elegant two-step synthesis of pyrrole-fused 1,3-diazepines has been reported by Liang et al. [42]. Firstly, a one-pot three components reaction involving ethyl cyanoformate (**92**), thiophenol (**93**), and cyclic ketones **94** afforded the piperidyl 3-oxazolin-5-one **95** under MW-activation at 80 °C, in the presence of TiCl_4_ and BF_3_·Et_2_O as Lewis acid catalysts (Scheme 15). By a marked contrast with a conventional heating, the MW-activation of this one-pot reaction allowed shortened reaction times (from 36 h to only 15 min) and improved conversion rates (around 60% under MW-irradiation).

**Scheme 16:**
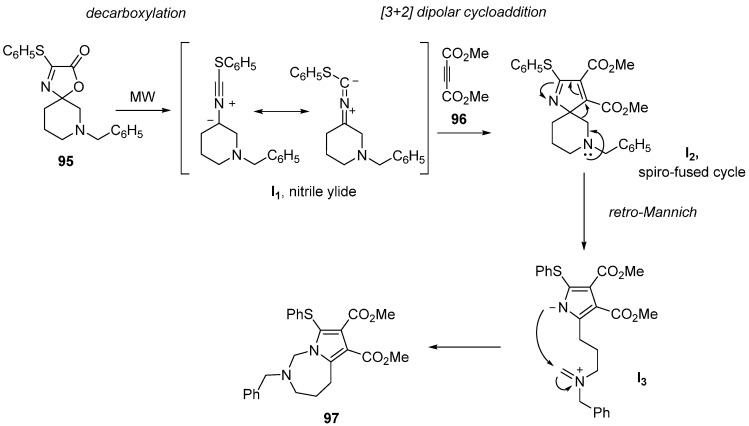
Mechanistic study of the pyrrole-fused 1,3-diazepines synthesis reported Liang et al. [42].

In the second step, the oxazolinone **95** is decarboxylated under MW-irradiation at 150 °C, resulting in the formation of a nitrile ylide (**I_1_**), which is trapped by a dipolarophile **96**, by means of a [3 + 2] dipolar cycloaddition, leading to a transient spiro-fused cycle (**I_2_**). This spiro intermediate is unstable, and a spontaneous retro-Mannich reaction leads in two steps to the pyrrolo[1,3]diazepine **97**. This unprecedented domino-process under MW-activation has been exemplified using series of various electron-deficient alkynes as dipolarophiles (Scheme 16).

**Scheme 17:**
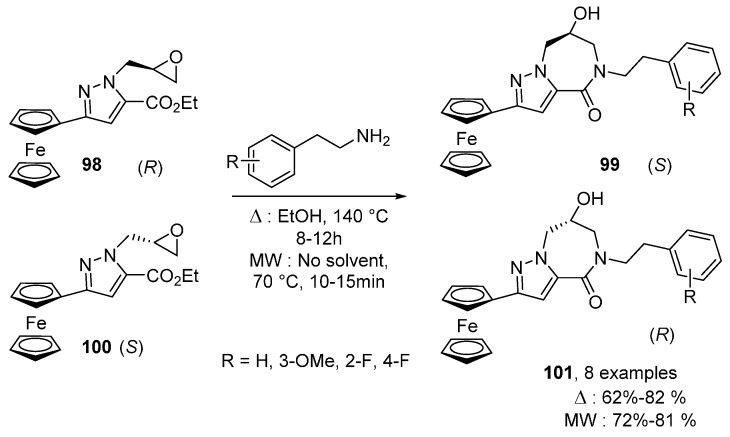
MW-synthesis of 2-ferrocenyl-7-hydroxy-5-phenethyl-5,6,7,8-tetrahydro-4*H*-pyrazolo[1,5-*a*][1,4]diazepin-4-ones.

A very useful synthesis of novel 2-ferrocenyl-7-hydroxy-5-phenethyl-5,6,7,8-tetrahydro-4*H*-pyrazolo[1,5-*a*][1,4]diazepin-4-one derivatives **99** and **101** has been described by Shen et al. [43] (Scheme 17). These compounds have been synthesized through the reaction of (*R*) or (*S*)-3-ferrocenyl-1-(oxiran-2-ylmethyl)-1*H*-pyrazole-5-carboxylates **98**, **100**, and substituted phenylethylamines. The reactions were performed under classical heating (in a sealed vessel) and MW-assisted heating (Scheme 17). MW-assisted solvent-free conditions provided the expected products with higher yields and shortened reaction times, related to the conventional ones [44].

Cellular assays on selected human lung cancer cell lines showed that some of these newly synthesized compounds exerted anti-proliferative activity against A549, H322 and H1299. In addition, it appeared that the anti-cancer activity wasn’t dependant of the chirality of the compounds [43,44].

##### Benzodiazepines and Their Fused Analogues

**Scheme 18:**
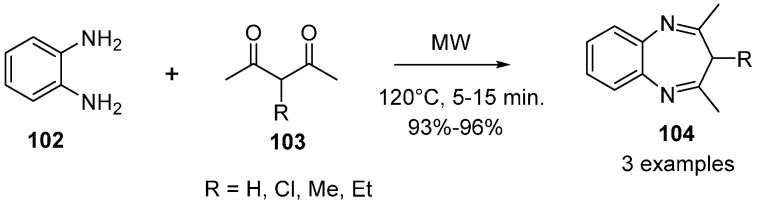
Green synthesis of 1,4-benzodiazepines.

The first MW-assisted synthesis of benzodiazepine derivatives through the condensation of 1,2-phenylenediamine and ethyl acetoacetate was reported in 1994 by the Soufiaoui group [45]. This synthesis has been extensively studied [46,47] and among the newest optimizations, Vaddula and co-workers reported a MW-assisted solvent- and catalyst-free green procedure (Scheme 18), which results in the formation of a series of 1,4-benzodiazepines **104** [48]. This condensation consists on mixing *o*-phenylendiamine **102** and substituted β-diketones **103** to afford quantitatively in shortened reaction times the required benzodiazepines (yields up to 95%). However, this reaction failed when using β-ketoesters or 3-chloropentane-2,4-dione.

**Scheme 19:**
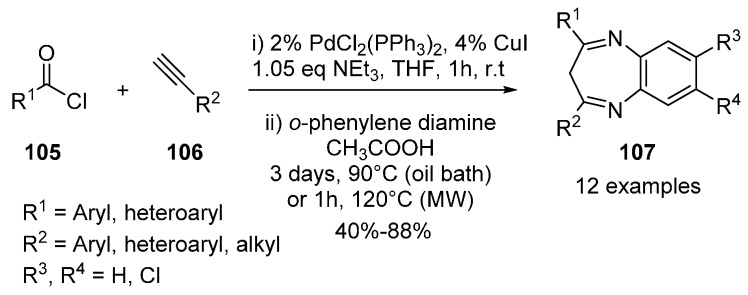
One-pot three-component reaction sequence leading to 2,4-disubstituted benzodiazepines.

Willy and co-workers [49] have synthesized 2,4-disubstituted benzodiazepines **107** in good yields from acyl chlorides **105**, alkynes **106**, and benzene-1,2-diamines (*o*-phenylenediamines) by a consecutive one-pot, three-component Sonogashira coupling/Michael addition/cyclocondensation sequence. These diazepines display intense solid-state fluorescence, but only weak emission in solution at room temperature. The authors reported that heating under MW-irradiation (120 °C) led to expected products in shortened reaction times (60 min) and better yields (40%–88%) compared to conventional reaction conditions, wherein the same products were obtained at 90 °C in 16 h (Scheme 19).

Multicomponent reactions (MCRs) have been widely studied for the synthesis of complex molecules. The use of a fluorine component as the limiting agent to stop the MCRs is a straightforward strategy to improve the final purification: for example, the condensed products could be recovered from the reaction mixture by using fluorine solid phase extraction (F-SPE). In addition, the fluorine could be used in a further step for compound’s derivatization to build elaborated multi-functionalized scaffolds.

**Scheme 20:**
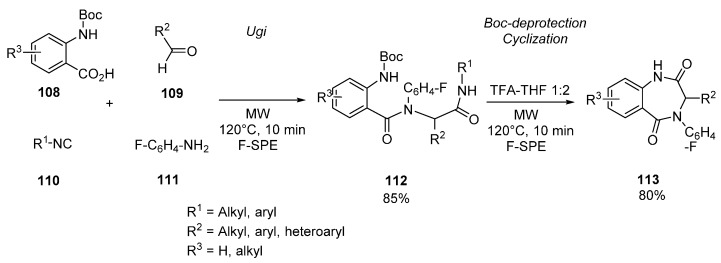
Two step synthesis of benzodiazepinones via an Ugi 4-components reaction and intramolecular cyclization.

The Ugi reaction is one of the most famous examples of MCRs. This reaction consists in mixing acid **108**, aldehyde **109**, isocyanide **110** and aniline **111**, and leads to pseudopeptidic moieties **112**. In this context, Zhang et al. [50] reported a two-step synthesis of benzodiazepinones **113** as illustrated in Scheme 20. The protocol occurs through an Ugi reaction under MW-irradiation, followed by a cyclization in acidic media, which also proceeds under MW-irradiation. Final products are obtained in approximately 70% yield (two steps, Scheme 20).

**Scheme 21:**
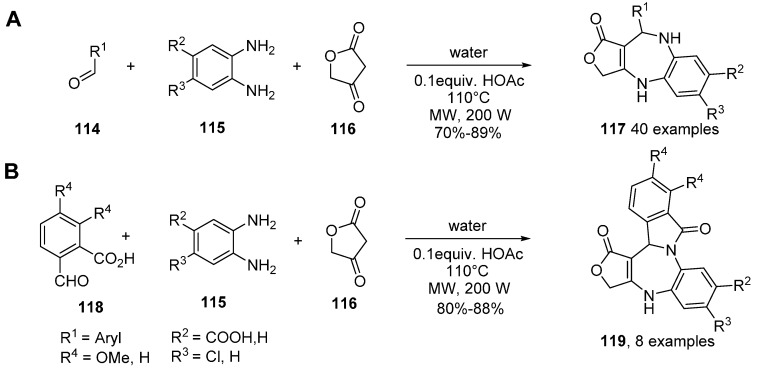
Synthesis of benzo[*f*]azulen-1-ones and isoindole-fused furo[1,4]diazepines in aqueous media.

Moreover, Wang et al. [51] reported another green chemistry procedure affording benzo[*f*]azulen-1-one derivatives **117**. This reaction is achieved by reacting benzene-1,2-diamines **115**, tetronic acid **116**, and various aldehydes **114** in aqueous media, under MW-irradiation, and does not require the use of a metal catalyst (Scheme 21A). The products have been obtained within 12-15 min in good to excellent yields, and with excellent regioselectivity. Indeed, only one single regioisomer was generated when 3,4-diaminobenzoic acid and 4-chlorobenzene-1,2-diamine were employed as substrates.

To extend the scope of this reaction, functionalized 2-formylbenzoïc acid derivatives **118** have been mixed with diamines **115** and tetronic acid (**116**) (Scheme 21B). In this case, the authors observed formation of pentacyclic isoindole-fused furo[1,4]diazepines **119.** The unprecedented regioselective reaction occurred in excellent yields (up to 81%) and in shortened reaction times (less than 25 min).

To summarize, this green chemistry procedure displaying several attractive characteristics, such as the use of water as solvent, concise conditions, short reaction time, easy work-up, and reduced side-products formation; overall, this synthesis proceeds without strong acidic and/or metal as catalysts.

**Scheme 22:**
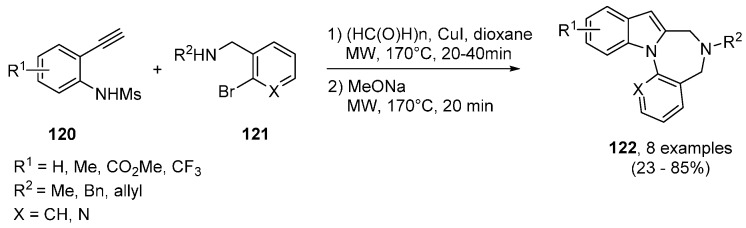
Cu(I)-catalysed domino three-component reaction leading to indole-fused 1,4-diazepines.

**Scheme 23:**
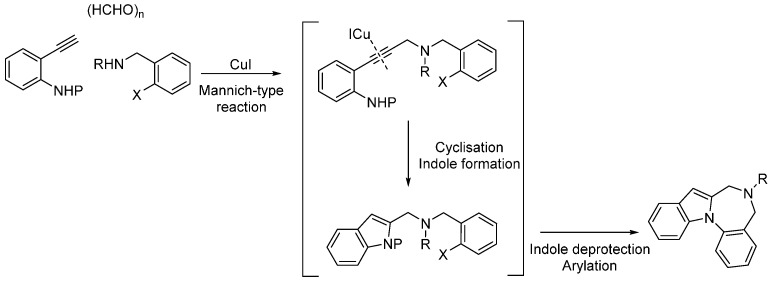
Mechanistic study of the indole-fused 1,4-diazepine synthesis reported by Fujii and Ohno [52].

The groups of Fujii and Ohno from Kyoto University have developed a Cu(I)-catalysed domino three-component coupling/indole formation/*N*-arylation cascade for the synthesis of indole-fused 1,4-diazepines **122** (Scheme 22) [52]. The first step consists on a Mannich-type reaction involving 2-ethynylanilines **120**, *O*-bromobenzylamines **121**, and paraformaldehyde; in the next steps, the indole ring is cyclized, and the subsequent mesyl deprotection followed by the *N*-arylation leads to the expected compounds (Scheme 23). In addition to the fused benzodiazepines, 1,4-diazepines fused with a pyridine or thiophene have been synthesized through the same protocol.

**Figure 8:**
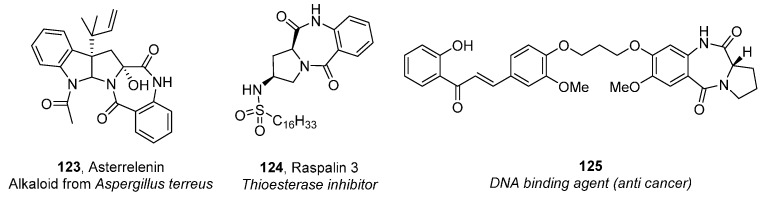
Relevant examples of bioactive pyrrolo[1,4]benzodiazepine-2,5-diones.

The pyrrolo[1,4]benzodiazepine-2,5-dione scaffold is also relevant in terms of pharmacological activity, as underlined by molecules such as asterrelenin (**123**), raspalin 3 (**124**) or a DNA binding fused compound **125** (Figure 8).

**Scheme 24:**
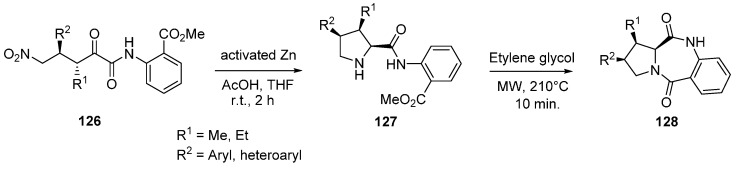
Benzodiazepinone synthesis through a reduction-double cyclization sequence of 1,3-ketonamides.

A very elegant stereoselective synthesis of pyrrolo[1,4]benzodiazepine-2,5-diones has been recently reported by Rodriguez’s group [53] (Scheme 24). This procedure is based on a reduction-double cyclization sequence of a 1,3-ketonamide motive **126**: the pyrrolidine intermediate **127** is obtained with a remarkable degree of optical purity (diastereomeric ratio (dr) 15:1) using a mixture of activated zinc in acetic acid as diastereoselective reductive agent. In the next step, the benzodiazepinone synthesis occurs through an intramolecular MW-activated lactamization. Even if the conversion rates remain modest (around 50%), the required final compounds **128** are isolated with a good enantiomeric excess (86% ee).

##### Seven-Membered Heterocycles with Two Distinct Heteroatoms

###### Benzothiazepines, Benzothiepines and Their Derivatives

**Figure 9:**
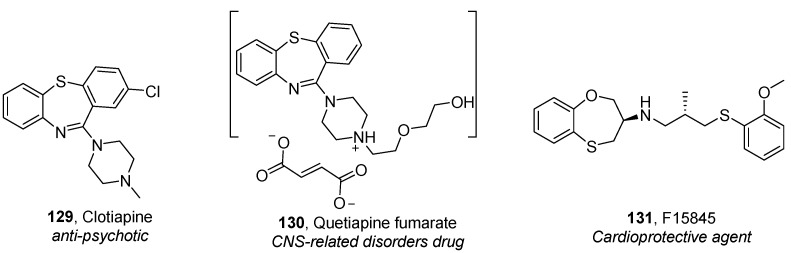
Relevant examples of bioactive dibenzothiazepines.

Several dibenzothiazepine derivatives have been marketed for their therapeutic uses. In this class of compounds, the two leading drugs are clotiapine (**129**), an anti-psychotic agent, and quetiapine fumarate (**130**), which is used in the treatment of central nervous system disorders (Figure 9) [54]. Another compound of interest is F15845 (**131**); this benzoxathiepine is a blocker of persistent sodium levels and is active in vivo to prevent ischemia and angina [55].

**Scheme 25:**
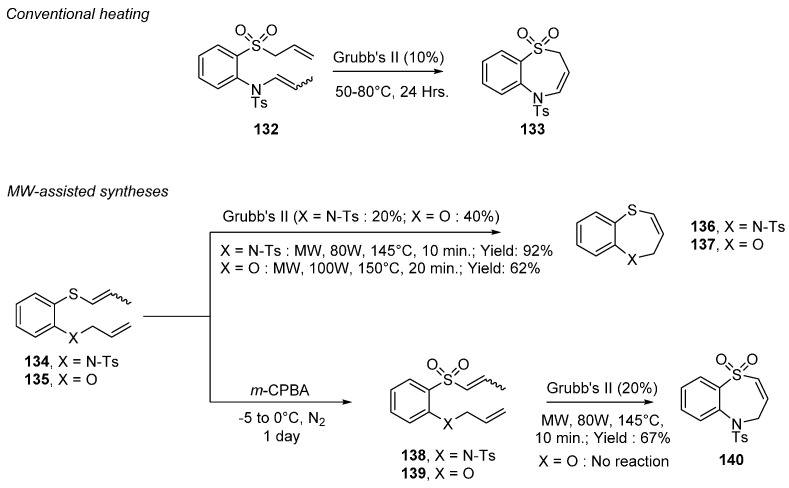
MW-assisted RCM affording benzo-fused dibenzothiazepines and dibenzoxathiepines.

Different strategies have been used to synthesize dibenzothiazepines. For example, the ring closure metathesis (RCM), which is one of the most popular methodologies leading to heterocycles, has been used by Yadav and co-workers [56] for the preparation of series of seven-membered, benzo-fused dibenzothiazepines and dibenzoxathiepine (Scheme 25).

In this strategy, a conventional RCM procedure, using Grubb’s II catalyst, afforded 2,5-dihydro-1,5-benzothazepine **133** in moderate yields (60%) and good regioselectivity, although the reaction was very slow (approximately 24 h). Conversely, the synthesis of the vinyl sulfide and sulfoxide analogs (4,5-dihydro-1,5-benzothazepines **136** and **140**), does not occur under conventional heating, and requires a MW-activation and a larger amount of catalysts.

In addition, using mercaptophenol (**135**) as starting material led to 2*H*-1,5-benzoxathiepine **137** under MW irradiation in good yields (62%). However, using this sulfoxide, and despite the use of harsh MW-assisted conditions, including high temperatures, long reaction times and large amount of Grubb’s II catalyst, the authors were unable to synthesize the sulfoxide analog of compound **139**.

**Scheme 26:**
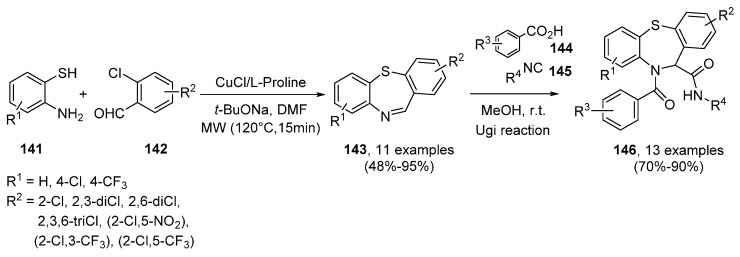
Copper-assisted C-S bond formation leading to series of dihydrodibenzo[*b*,*f*][1,4]-thiazepine-11-carboxamides.

**Scheme 27:**
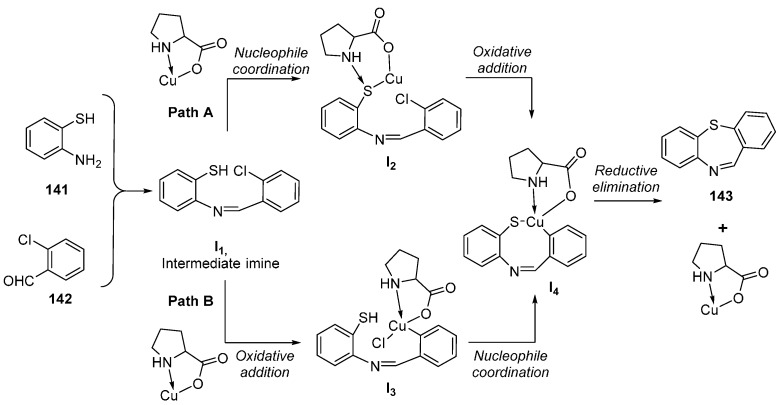
Mechanistic study of the C-S bond formation reported by Saha et al. [57].

Saha et al. [57] have reported a two-step MW-assisted synthesis of a series of dihydrodibenzo[*b*,*f*][1,4]-thiazepine-11-carboxamides (Scheme 26). The cyclisation step consists on a reaction between aminothiophenols **141** and *o*-chlorobenzaldehydes **142**, leading to the intermediates **143**. In the first step of this synthesis, amine and aldehydes react together, and afford the imine intermediate **I1** (Scheme 27) according to a conventional base-catalyzed mechanism; in the second step, the seven-membered benzothiazepines **143** are cyclized through a copper assisted C-S bond formation. Under MW-activation, this reaction requires 10 mol % of catalyst and 20 mol % of l-proline as ligand and occurs in 15 min.

The mechanistic study of the C-S bond formation revealed two possible catalytic pathways. In the first one (Scheme 27, Path A), the coordination of the proline ligated Cu(I) complex to deprotonated thiolate (nucleophilic coordination, intermediate **I_2_**) precedes the oxidative addition. Conversely, in the second pathway (Scheme 27, Path B), the coordination to the thiolate occurs after the oxidative addition of the copper complex to the halogenobenzene (intermediate **I_3_**). 

The second step of the benzothiazepine carboxamides synthesis consists on an Ugi multi-components reaction involving the macrocycles **143**, aliphatic and/or aromatic carboxylic acids **144** and nitriles **145**. These Ugi reactions are managed under conventional activation, and afforded the various dihydrodibenzo[*b*,*f*][1,4]-thiazepine-11-carboxamide derivatives **146** in high yields.

**Scheme 28:**
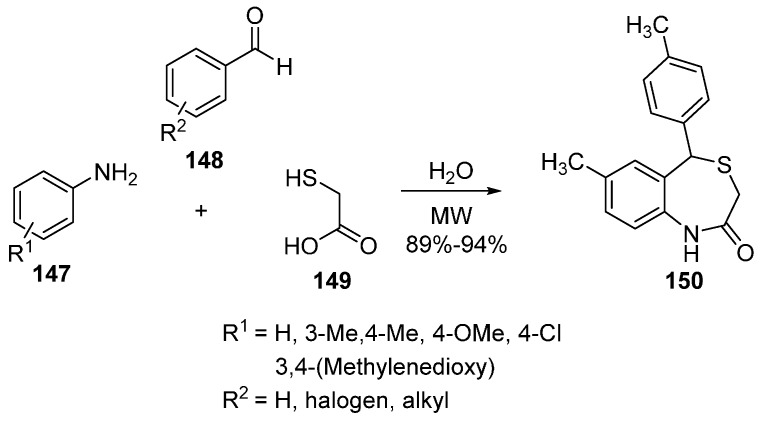
MW-assisted three-component reaction in water affording benzo[*e*][1,4]thiazepin-2(1*H*,3*H*,5*H*)-ones.

Tu et al. [58] have reported a straightforward strategy for the synthesis of benzo[*e*][1,4]thiazepin-2(1*H*,3*H*,5*H*)-ones **150**, via a MW-assisted multi-component reaction in aqueous media, yielding to the expected molecules with excellent conversion rates (89%–94%) and shortened reaction times (7–10 min). This green strategy consists on a three-component reaction involving various aromatic amines **147**, series of aldehydes **148**, and mercaptoacetic acid (**149**, Scheme 28).

**Scheme 29:**
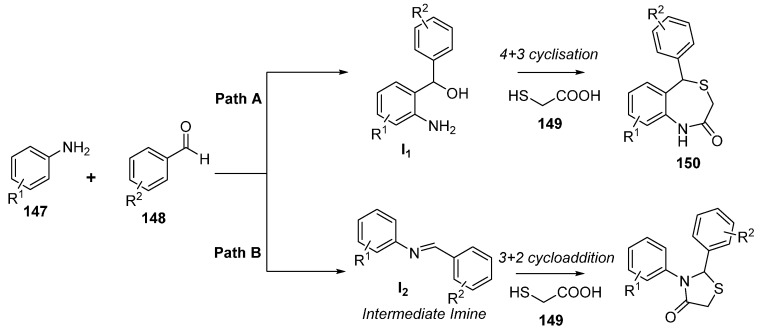
Mechanistic study of the benzo[*e*][1,4]thiazepin-2(1*H*,3*H*,5*H*)-ones syntheses reported by Tu et al. [58].

Interestingly, the regioselectivity of this macrocyclisation depends on the nature of the aldehyde’s substituent (group R^2^ on compounds **148**). Indeed, electron-donating groups act in favour of an aromatic electrophile substitution (Scheme 29, Path A), resulting in the formation of intermediate **I_1_**. In the second step, this intermediate **I_1_** undergoes a [4 + 3] cyclisation with mercaptoacetic acid, affording the seven-membered lactam **150**. Conversely, electron-withdrawing groups lead to a conventional nucleophilic attack of the carbonyl, resulting in the formation of the intermediate imine **I_2_**, whose [3 + 2] cycloaddition with mercaptoacetic acid gives the corresponding thiazolidinone (Scheme 29, Path B). Nevertheless, the presence of a potent electron-donating group on the aromatic amine can overcome the chemoselectivity, and subsequently restore the Path A, and the synthesis of the seven-membered ring.

**Scheme 30:**
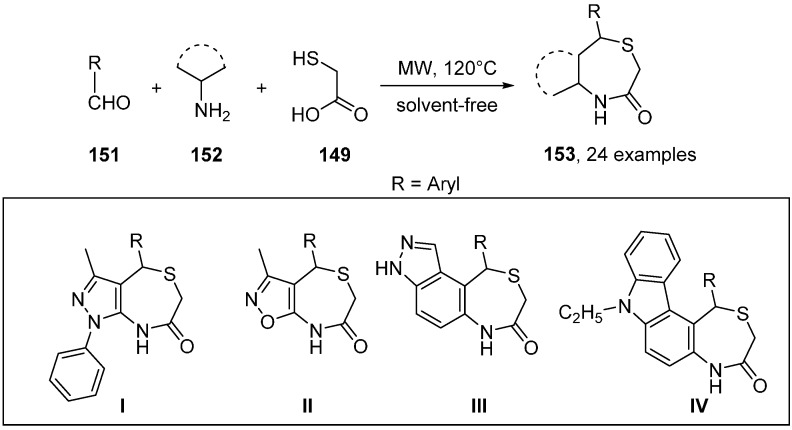
Solvent-free multicomponent reaction affording 1,4-thiazepines.

A straightforward solvent-free MW-assisted multicomponent synthesis of 1,4-thiazepines fused with carbazoles, pyrazoles or isoxazoles has been reported by Shi et al. [59]. This strategy illustrates the potency of the multicomponent reactions as a tool for the “diversity-oriented syntheses”. These approaches not only allow the quick and efficient synthesis of heterocycle libraries, but also reduce concomitantly their environmental impact. Practically, the 1,4-thiazepines have been synthesized through MW-assisted condensation of aldehydes, mercaptoacetic acid and series of heteroaromatic amines. These syntheses were carried out in absence of solvent at 120 °C, and were completed in less than 10 min (Scheme 30). 

Among them, several derivatives exhibited in preliminary biological assays anti-oxidant and/or anti-proliferative activities. Indeed, at the dose of 2 mg/mL, these compounds reduce the HCT 116 cancer cells viability by 50%, without affecting healthy lymphocytes viability [59].

###### Oxazepines and Their Derivatives

**Figure 10:**
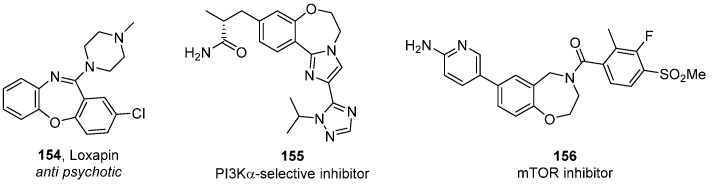
Examples of representative oxazepines.

The oxazepine core is a seven-membered heterocycle with one atom of nitrogen and one atom of oxygen. This scaffold is also used to design potent therapeutic agents, as illustrated by Loxapin, a marketed anti-psychotic drug; in addition, several oxazepines have been designed as potent kinase inhibitors (Figure 10) [60,61,62]. 

**Scheme 31:**
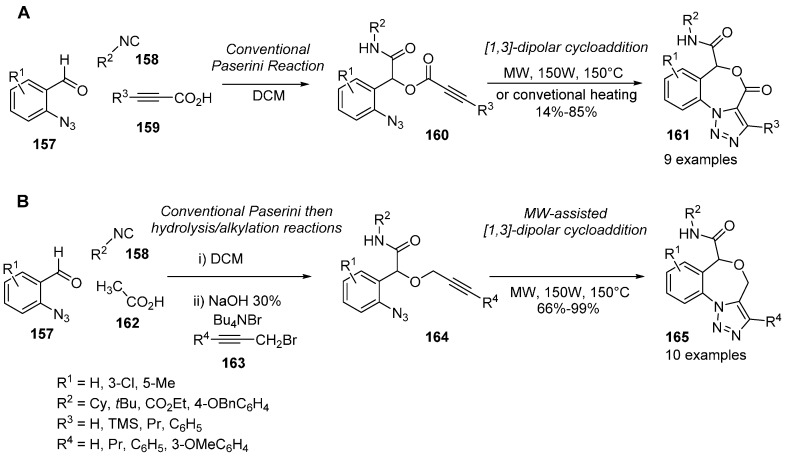
Synthetic route to triazolo-fused benzoxazepinones and benzoxazepines via successive Passerini reaction and [1,3]-dipolar cycloaddition.

An elegant synthesis of unprecedented triazolo-fused benzoxazepinones **161** (Scheme 31A) and benzoxazepines **166** (Scheme 31B), which combines a conventional 3-component Passerini reaction with a MW-assisted Hüisgen azide-alkyne [1,3]-dipolar cycloaddition has been reported by Basso and co-workers [63].

The dipolar cycloaddition leading to the final benzoxazepinones **161** could be performed either under conventional heating or under MW-irradiation. Interestingly, the conventional heating is very detrimental in terms of reaction speed (several days are required for a complete conversion, due to the poor reactivity of the alkyne group), but generally leads to cleaner products than the MW-assisted reaction. Indeed, when using the MW-assisted procedure, the authors failed to isolate the pure final benzoxazepinones **161**. In marked contrast, the cycloaddition leading to the final benzoxazepines **165** is easily achieved by means of MW-irradiation. 

**Scheme 32:**
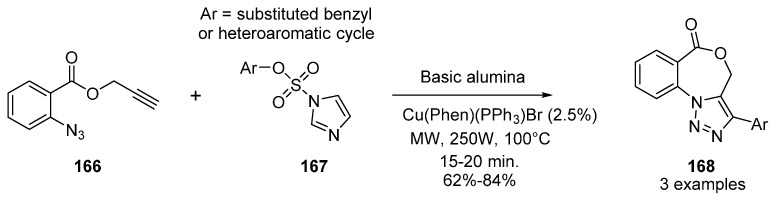
One-pot, green synthesis of triazolobenzoxazepines through Sonogashira azide-alkyne cycloadditions.

Also, a MW-assisted green synthesis of triazolobenzoxazepines has been suggested by Sen and co-workers [64]. This one-pot reaction is based on a Sonogashira azide-alkyne cycloaddition, and was carried out using Cu(Phen)(PPh_3_)Br as catalyst (Scheme 32). Overall, this synthesis is performed on a solid support, basic alumina, which has the ability to absorb organic compounds and transmit microwave irradiation without adsorbing or restricting it. In this context, the cyclisation occurs with only 2.5% of catalysts and affords in shortened reaction time the expected products in good yields (up to 60%). Moreover, the solid support can be re-used after washing three times without any changes in conversion rates. This green protocol of cyclization is compatible with a huge variety of scaffolds and can be used to build other heterocycles such as triazolobenzodiazepines or triazolo- benzoxazines. 

**Scheme 33:**
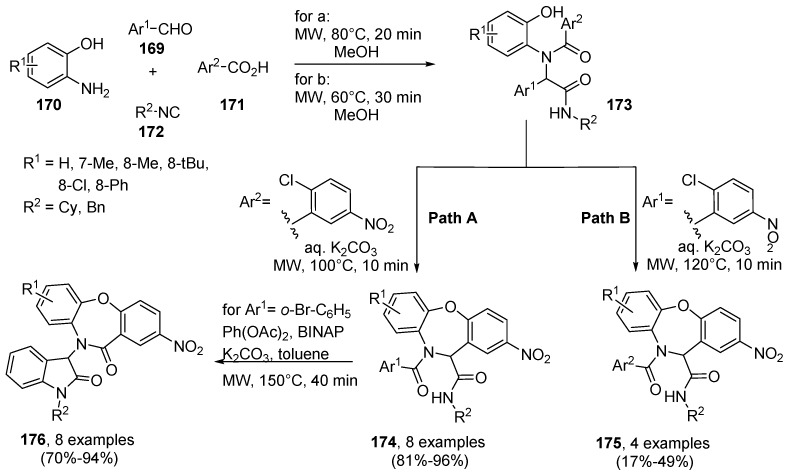
Oxazepines synthesis via Ugi 4-component reaction and intramolecular *O*-arylation.

The MW-assisted synthesis of highly functionalized dibenz[*b*,*f*][1,4]oxazepin-*11*(*10H*)-ones **175** and **176** has been also reported by Xing et al. [65] (Scheme 33). The oxazepine derivatives were obtained via a one-pot two-reaction sequence, which combines an Ugi reaction and an intramolecular *o*-arylation, in good to excellent yields.

#### 2.1.3. Seven-Membered Heterocycles with Three Heteroatoms

##### Benzotriazepines and Their Fused Analogues

**Figure 11:**
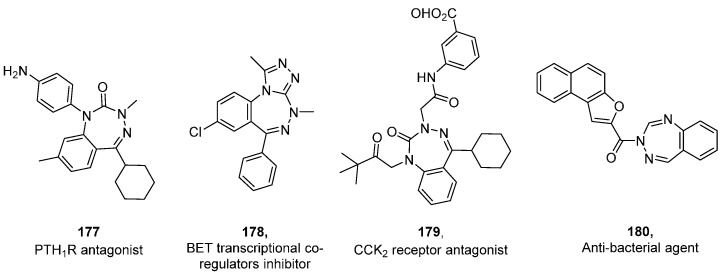
Representative examples of bioactive benzotriazepines.

Benzotriazepine derivatives are commonly used for their antibacterial, antiviral, anti-protozoan (*plasmodium*) and psychotropic activities; series of benzotriazepines have been also designed as potential anti-cancer or anti-viral agents [66,67,68,69] (Figure 11). 

**Scheme 34:**
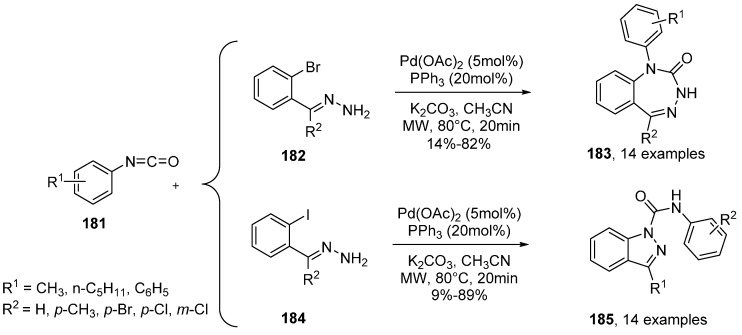
Synthesis of benzotriazepines and indazoles.

In addition, some of them have been also used as acaricidal, herbicidal and insecticidal agents. In this context, Dong et al. [70] reported an interesting synthesis of benzotriazepines based on a palladium-catalysed cyclization of aryl isocyanates **181** and phenyl hydrazones under MW-irradiation (Scheme 34). Taking advantage of the difference between iodine and bromine in term of reactivity towards the Pd(0) oxidative addition, the authors have reported a divergent synthesis of benzotriazepines **183** and 1-arylamide-1*H*-indazoles **185**. Indeed, bromophenyl hydrazones **182** afforded exclusively the benzotriazepines derivatives **183** with 5 mol % of Pd(OAc)_2_ and 10 mol % of PPh_3_ in 20 min and under MW-activation with high conversion rates, whereas in the same conditions iodine hydrazones **184** lead to the 1-arylamide-1*H*-indazole series **185**. It is noteworthy that in this latter case, the use of bulky derivatives as starting material lead to a mixture of benzotriazepines and indazoles.

**Scheme 35:**
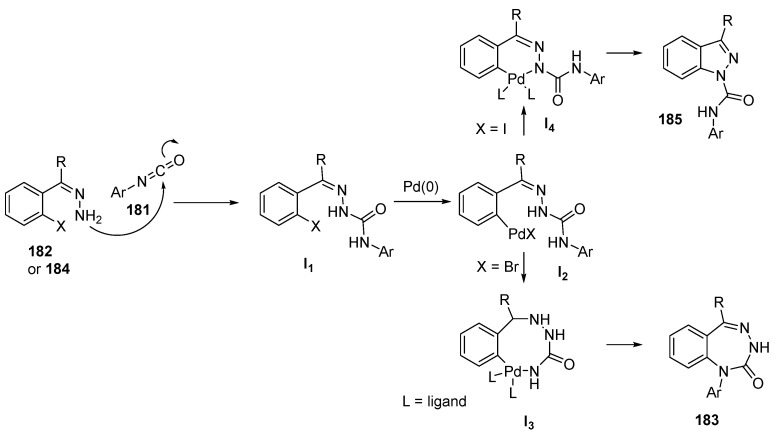
Mechanistic study of the benzotriazepines and indazoles synthesis reported Dong et al. [70].

The authors hypothesized the mechanism outlined in Scheme 35. Indeed, they postulated that the regioselectivity of the reaction is driven by the intramolecular palladation during the second phase of the reaction, and depends: (i) on the nature of the halogen; and (ii) on the steric hindrance of the aryl hydrazones.

**Scheme 36:**
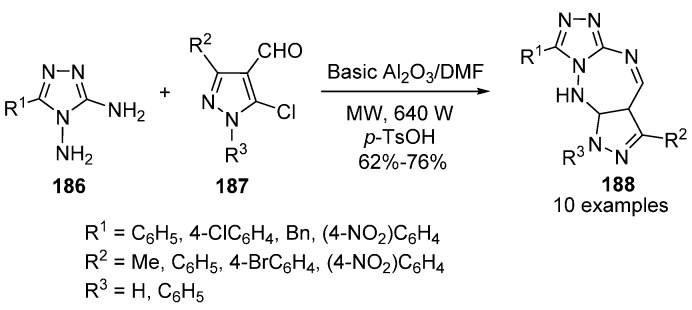
Green procedure for the synthesis of fused triazepines on a solid support.

Gupta et al. [71] have reported a simple, efficient and green MW-assisted procedure for the synthesis of 1-substituted-8-aryl-3-alkyl/aryl-4*H*-pyrazolo[4,5-*f*][1,2,4]triazolo[4,3-*b*][1,2,4]triazepines **189**. This reaction occurs on a solid support (basic alumina), using DMF as an energy transfer agent, and *p*-TsOH as catalyst. The products have been obtained in good yields (62%–76%) with high purity, in 4 to 25 min (Scheme 36). Lower conversion rates were obtained when conventional heating was used.

**Scheme 37:**
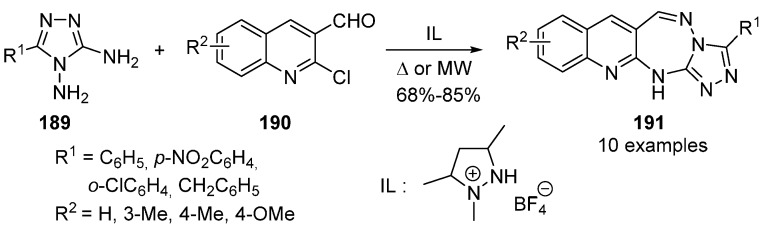
Green procedure for 1,2,4-triazepine synthesis in ionic liquid as solvent.

In a similar way, these authors [72] also reported the synthesis of the 1,2,4-triazepine analogs **192** according to an original green procedure involving an ionic liquid (IL) as solvent, and using either MW-activation or oil-bath heating. The products were isolated in good to moderate yields, and in high degrees of purity (Scheme 37). Moreover, the ionic liquid was found to be recyclable for at least three consecutive reaction cycles, making the process cost-effective and economic. In a preliminary anti-fungal assay, some compounds showed high potencies against *Aspergillus niger* and *Pencillium notatum*, *Aspergillus flavus* and *Rhizopus*.

##### Thiadiazepines and Benzothiadiazepines

**Scheme 38:**
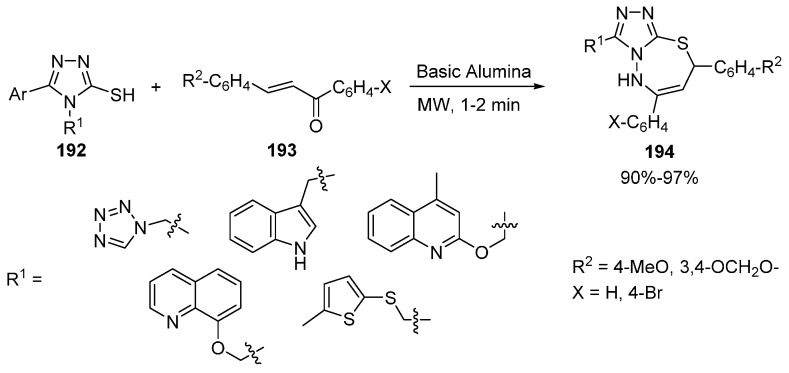
Solvent free synthesis of thiadiazepines.

A solvent-free and MW-assisted procedure has also been developed for the synthesis of potentially bioactive thiadiazepines **194**. This procedure involved the reaction of 1-amino-2-mercapto-5-substituted triazoles **192** and substituted chalcones **193** using basic alumina as solid support (Scheme 38). Interestingly, this reaction requires longer reaction times and exhibits lower conversion rates when performed in solution phase [73]. Recently, *p*-TsOH has been also reported as a suitable catalyst for this transformation under MW-irradiation [74].

**Scheme 39:**
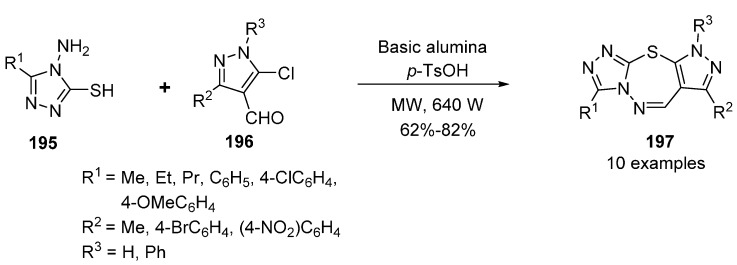
Green synthesis of thiadiazepines.

To the best of our knowledge, very few MW-induced syntheses of benzothiadiazepines on solid support have been reported. In this context, the simple, efficient and green procedure highlighted by Gupta and co-workers is a remarkable exception [75]. In this study, the authors demonstrated that the reaction of 1-amino-2-mercapto-1,3,4-triazoles **195** with 5-chloro-4-formyl-1,2-pyrazoles **196** in presence of *p-*TsOH and basic alumina, leads the expected highly functionalized [1,3,4]thiadiazepines **197** in less than 10 min and with good yields (up to 62%). The basic alumina acts as a solid support; it increases not only the sulfur atom basicity, but also provides an environment-friendly media. Lastly, *p-*TsOH converts carbonyl into a potent leaving group and therefore increases the reaction speed (Scheme 39).

**Scheme 40:**
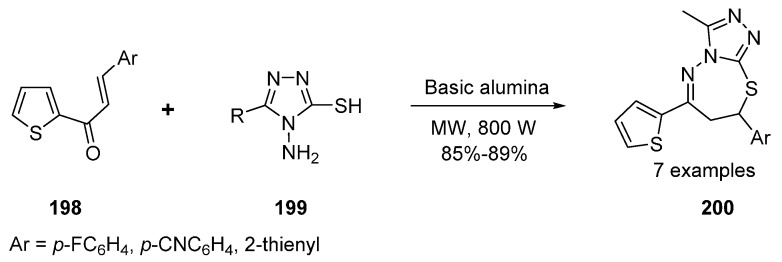
Green synthesis and solvent free synthesis of thiadiazepines.

Lastly, Assaker and co-workers reported a green protocol and solvent free synthesis of [1,3,4]-thiadiazepines (Scheme 40) [76]. To this end, chalcones **198** were allowed to react with a series of 1-amino-2-mercapto-5-substituted triazoles **199** in presence of basic alumina and under MW-irradiation, to afford the expected 7,8-dihydro-3,7-diaryl-9-(thiopen-2-yl)-[1,2,4]triazolo[3,4-*b*][1,3,4]thiadiazepine derivatives **200**. These compounds have been obtained with high yields (up to 89%) and short reaction times (less than 30 min). By a marked contrast, a conventional heating (refluxing acetone, K_2_CO_3_ as base) afforded the products with moderate yields and long reaction times (more than 15 h).

### 2.2. Synthesis of Other Macroheterocycles

#### 2.2.1. Cyclic Peptides and Pseudo-Peptides: MW-Irradiation Applied to SPPS

The MW-irradiation procedure applied to manual or automated solid-phase peptide synthesis (SPPS) for the coupling reaction and/or for the N^α^-deprotection, has been extensively studied and used in the last decade. Compared to conventional SPPS, this methodology results in gains on final peptide yields, especially for peptides with long sequences or peptides including “difficult” sequences (such as β-sheet type structures), combined with an increase of the crude peptide purity [77]. Moreover, the MW-activation is not detrimental in terms of optical purity: the racemization rates are usually very close when heating at the same temperature under MW-activation or when using a conventional heating. Recent studies have demonstrated that these improvements are reliable to the MW thermic effects rather than to the specific electromagnetic effect. Lastly, MW-assisted SPPS is compatible with several solid supports, such as polystyrene resins, PEG supports, Wang-resin, chlorotrityl resins, and rink amide resins [78,79,80]. 

In this context, MW-assisted SPPS has been applied to the macrocyclization of peptides, pseudo-peptides and peptidomimetics (peptoids, β-peptides). To this end, different cyclization strategies have been reported, among them thioether formation, lactamisation, RCM, or Glaser coupling.

**Scheme 41:**
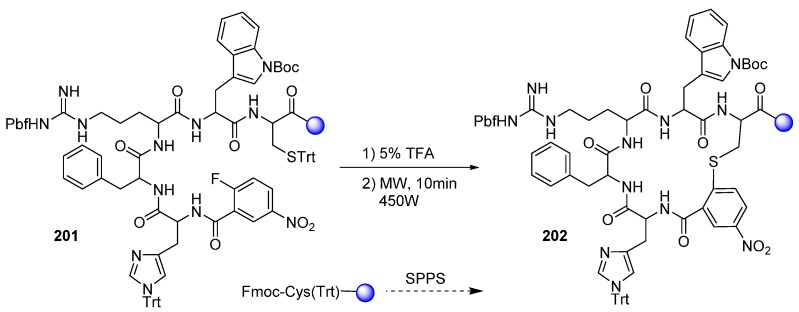
MW-assisted synthesis of 20-membered cyclic peptidomimetics.

For example, a series of 20-membered macrocyclic peptidomimetics has been recently synthesized using MW-irradiation for the cyclization step [81]. This MW-assisted cyclization occurs through a nucleophilic aromatic substitution (S_N_Ar) between a cysteine and a *p*-fluoronitrobenzene derivative before the cleavage of the peptide from the resin. This cyclization is performed at 50 °C and within 10 min only (Scheme 41). This straightforward synthesis afforded the macrocyclic peptide **202** in high yield (70%) and with high degree of purity. 

**Scheme 42:**
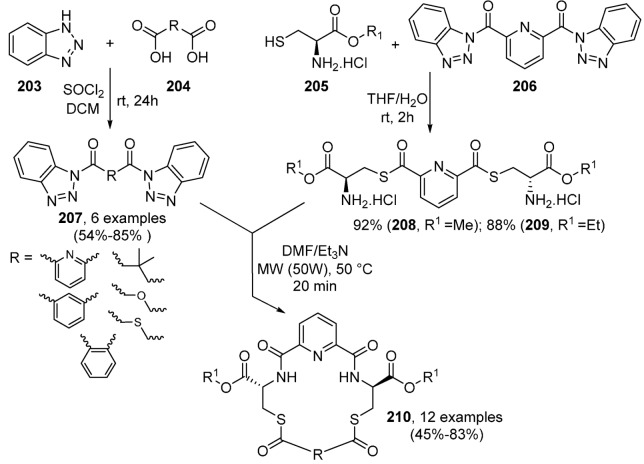
Example of peptidomimetic cyclization through a MW-assisted thioester bond formation.

Another interesting example of peptidomimetic cyclization through a MW-assisted thioether bond formation has been recently reported by Ibrahim et al. [82]. Firstly, the *N*-acylbisbenzotriazoles **207** were obtained under classical conditions by mixing appropriate dicarboxylic acids **204** and 1*H*-benzotriazole **203** in the presence of thionyl chloride (Scheme 42). In the next step, these derivatives were converted in excellent yields into bis(*S*-acylcysteine) esters by means of a regioselective *S*-acylation of cysteine esters. The resulting pyridine dicysteine ester hydrochlorides **208**, **209** were subsequently treated with *N*-acylbisbenzotriazoles **207** in the presence of triethylamine under MW-irradiation for 20 min to afford the cyclic and enantiopure peptidomimetics **210** via double *N*-acylation in moderate to good yields (Scheme 42).

Some of these cyclic peptidomimetics, exhibited potent minimum inhibitory concentration (MIC) against *C. albicans* ranking between 0.0075 µg/mL and 0.03 µg/mL, by comparison with the reference drug amphotericin B, which showed in the same assay a MIC = 0.35 µg/mL. Moreover, the best hit compound is 10,000-fold more potent against *K. pneumoniae* than the reference compound ciprofloxacin. Conversely, in a cytotoxic assay, these molecules exhibited moderate activities against a panel of several human cancer cell lines by comparison with doxorubicin used as reference [82]. 

**Scheme 43:**
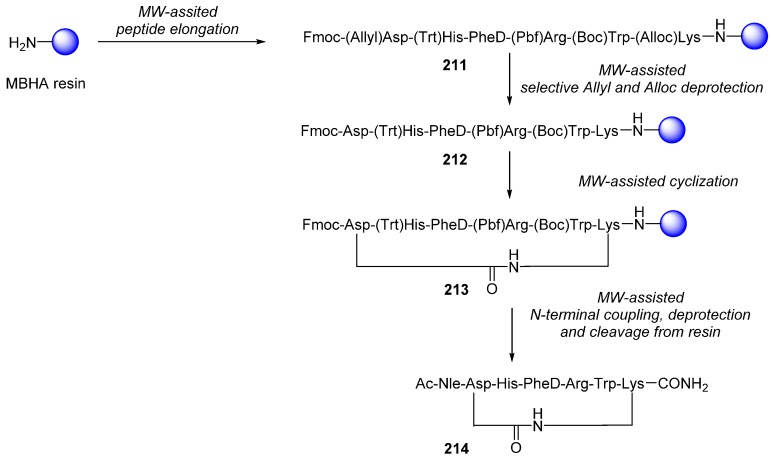
MW-assisted SPPS and on-resin cyclization of 7-mer peptides.

Tala and co-workers have recently reported a MW-assisted SPPS of two 7-mer peptides cyclized through a side-chain lactam bridge (Scheme 43) [83]. By a marked contrast with conventional methods leading to lactam bridged peptides, and which involve special reaction conditions, specific protecting groups, require long reaction times and lead sometimes to huge levels of side products, the process reported by these authors afforded the cyclized peptides with a high level of purity and in shortened reaction times. In addition, to simplify the purification, the cyclization step is performed while the peptide is still bound to the resin.

It is important to note that all steps of this synthesis occurred under MW-activation, from the peptide elongation on the rink amide MBHA resin to the cleavage of the final product. The coupling steps occurred using a standard HBTU/DIEA method in only 5 min under MW irradiation (30 W, 75 °C); Fmoc deprotections were performed with 0.1 M HOBt in a mixture of piperidine and DMF under MW irradiation (30 W, 75 °C), leading to **211** in shortened reaction times.

Interestingly, under MW-irradiation (30 W, 75 °C), the concomitant and specific Allyl and Alloc deprotection of Lys and Asp has been completed in 2 min in an open vessel, using a mixture of PhSiH_3_ (20 eq.)/Pd(PPh_3_)_4_ (0.25 eq.). This experimental procedure is over-simplified by comparison with the conventional procedure, which proceeds in a sealed vessel, under neutral atmosphere and requires at least 1 h. The resulting peptide **212** is then cyclized in 10 min only under MW-irradiation (30 W, 75 °C) using HBTU as coupling agent. This process is also over-simplified, since the conventional lactamization procedure requires a mixture of HBTU/PyBOP/HATU, HOBt and DIEA and is usually completed at room temperature in 2–24 h. Finally, the last steps (*N*-terminal coupling, final deprotection and cleavage from the MBHA resin) occur under MW-irradiation and shortened reaction times. 

**Scheme 44:**
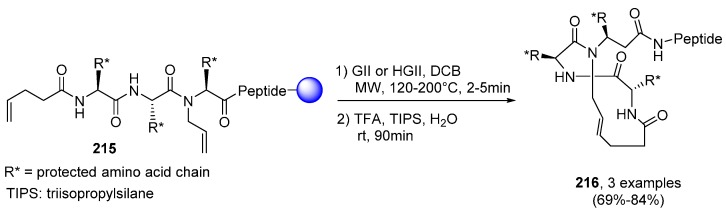
Example of peptide cyclization through MW-assisted RCM.

Another popular strategy used for the preparation of cyclic peptides consists of using a RCM step. In this context, Chapman et al. [84] reported an elegant solid-phase synthesis of hydrogen-bond surrogate (HBS) *α*-helices including a key RCM reaction (Scheme 44). In this artificial *α*-helice scaffold, the *N-*terminal H bond was replaced by a covalent C-C bond.

In this study, three different 13-membered macrocycles **216** were obtained in high yields. Interestingly, under MW-activation, warming at 120 °C for 2 min was required when Grubbs II was employed as catalyst, while warming at 200 °C for 5 min was required when using Hoveyda-Grubbs II catalyst. Importantly, both catalysts afforded the expected macrocycles in high yields compared with conventional heating wherein it showed to be inactive.

**Scheme 45:**
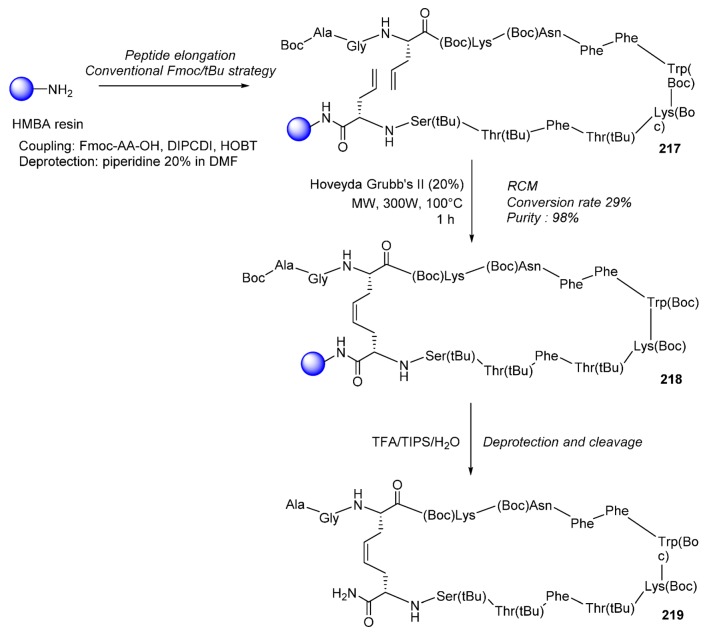
Synthesis of a 10-mer cyclic through on-resin MW-assisted RCM.

A similar RCM cyclization on an on-resin peptide has been recently used by Gago and co-workers, for the synthesis of a series of somatostatin analogues (Scheme 45) [85]. To compare the effects of the resin on the cyclization step, the peptides have been elongated on two types of resin (chlorotrityl resin and HMBA rink amide resin), according to a conventional Fmoc/*t*Bu strategy: the authors used a low substitution level on the resins, in order to avoid cross-metathesis. When using the chlorotrityl resin, the authors were unable to obtain a good yield for the cyclization after treatment with Grubb’s II catalyst under MW-irradiation (45 °C or 100 °C, 48 h). They hypothesized that this result is due to the poor stability and the strong lability of the chlorotrityl resin at high temperatures. However, in the case of the HMBA resin, the authors reported very good results. Indeed, they obtained the required somatostatin analogues with an excellent purity level (up to 98%) using the Hoveyda-Grubb’s II catalyst at 100 °C and without using a chaotropic agent (LiCl).

**Scheme 46:**
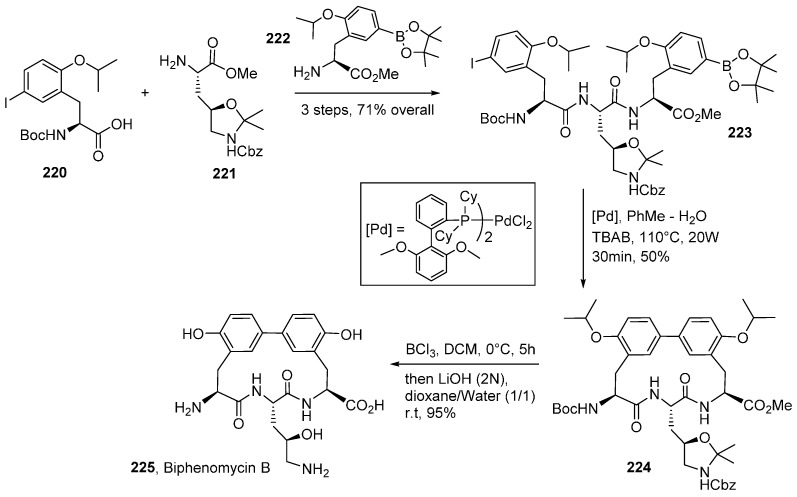
Synthesis of biphenomycin B via a Pd(0) C-C bond formation.

Biphenomycin B (**225**), a natural cyclic peptide with an unusual *endo* aryl-aryl bond, displays potent activities against Gram-positive β-lactam-resistant bacteria, and has been recently re-synthesized by Zhu et al. [86] (Scheme 46). The key step of this synthesis is a MW-assisted intramolecular Suzuki-Miyaura cross-coupling reaction involving a linear tripeptide **223**, which could be easily obtained from three non-proteinogenic amino acids **220**–**222**. After optimizations, the intramolecular Suzuki-Miyaura coupling afforded the desired macrocycle **225** in 50% yield.

**Scheme 47:**
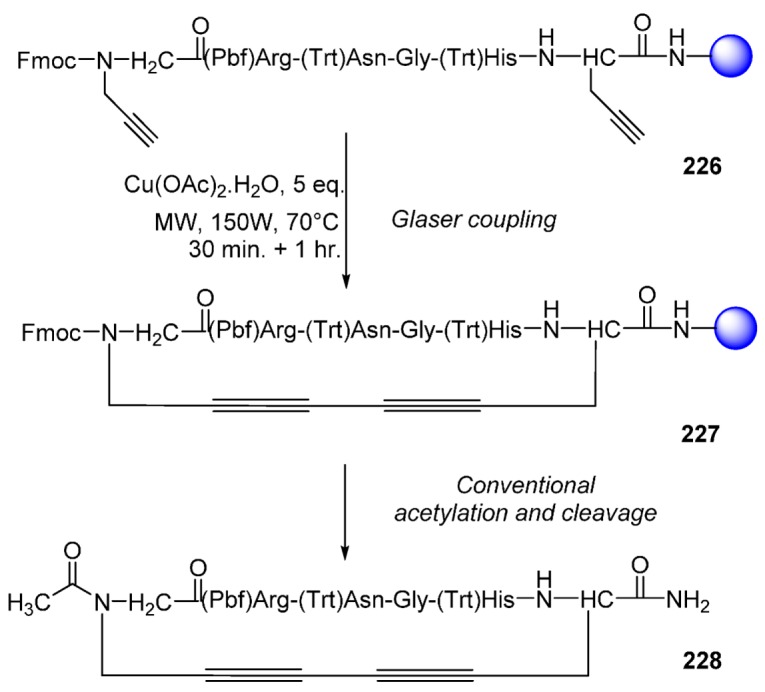
Peptide cyclization through an on-resin Glaser reaction.

Mallet and co-workers [87] have recently reported the supported synthesis of 1,3-butadiyne constraint cyclopeptides, which could be obtained through an intramolecular Glaser-Eglinton oxidative coupling managed under MW-irradiation. The Glaser coupling occurs between two terminal alkynes, is catalyzed by a Cu(I) salt (usually Cu(OAc)_2_), and proceeds under mild conditions in presence of a source of nitrogen (such as acetonitrile) and of oxygen (Scheme 47). 

In this study, the authors report an on-resin method (MBHA rink amide resin), using either propargylglycine or *N*-propargylglycine located at the two extremities of the peptidic sequence. They highlight the relevance of the MW-activation: indeed, by comparison with conventional heating, MW irradiation allowed shortened reaction times (from 16 h to less than 90 min), and afforded the cyclized peptides in better yields and higher purities (Scheme 47).

**Scheme 48:**
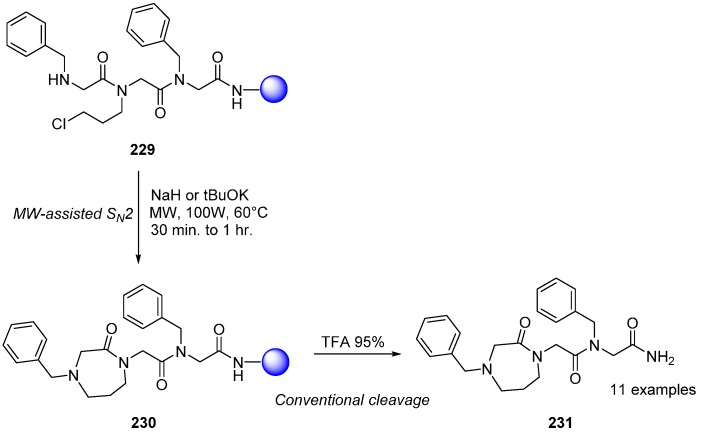
MW-assisted chain-to-tail cyclization of peptoïds.

An efficient synthesis of side chain-to-tail cyclization of peptoids through an S_N_2 reaction has been recently reported by Kaniraj and Maayan [88]. Peptoids form an important class of proteolytic-resistant peptidomemetics exhibiting an enhanced cell permeability. The conventional syntheses of chain-to-tail cyclic peptoids include click chemistry reactions, (triazole formation), lactam formation, triazine bridge synthesis and RCM. Each of them is generally efficient, but each of them requires the introduction of a specific functional group. Conversely, the strategy reported by Kaniraj and Maayan is a simple and versatile nucleophilic substitution which leads to the required cyclic peptoid in high yields and with an excellent degree of purity. It is to note that the MW-activation accelerates the reaction (from 4 days to 35 min) (Scheme 48).

#### 2.2.2. MW-Assisted Syntheses of Macrocyclic Natural Products and Analogues

**Scheme 49:**
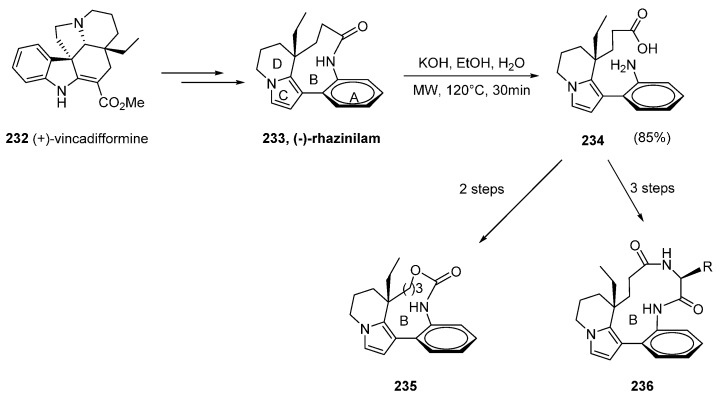
MW-assisted synthesis of (−)-rhazinilam derivatives.

(−)-Rhazinilam (**233**, Scheme 49) is a natural antimitotic compound isolated from *Rhazya stricta* (Apocynaceae) and from *Kopsia singapurensis*. This molecule exerts cytotoxic activity in vitro, since it inhibits microtubule assembly and disassembly and induces the abnormal formation of tubulin spirals; (−)-rhazinilam interacts with tubulin in the same range as colchicine (IC_50_ = 2 µM), but is less potent than vinblastine and taxol, two microtubule trafficking blockers. Moreover, (−)-rhazinilam appears to be inactive in vivo. These results prompted interesting structure-activity relationship studies. (−)-Rhazinilam can be described as an unusual tetracyclic motive, with an axially chiral phenyl (cycle A)-pyrrole (cycle C) subunit linked by a nine-membered lactam cycle (cycle B). Chemical and structural changes in the cycle B retain or enhance the drug’s affinity for the tubulin, whereas structural changes in the remaining three cycles induce a total loss of biological activity. Therefore, Baudoin’s group designed new (−)-rhazinilam derivatives in which the cycle B was enlarged **235**, **236** [89].

The corresponding syntheses are outlined in Scheme 49. The initial hydrolysis of the macrolactam (cycle B of rhazinilam) was performed on basic aqueous media, and led to compound **234** [89]. Interestingly, under conventional heating, this reaction requires harsh conditions (excess of KOH, refluxing mixture of water and ethanol, and one day of heating); by a marked contrast, this hydrolysis occurs in only 30 min and with 85% of yield under MW-irradiation (120 °C, 100 W, sealed tube). The following steps of this synthesis, among them the re-closing step, were performed under conventional procedures. The new series of (−)-rhazinilam derivatives include derivatives with 11- or 12-membered cycle B, which were closed through an endocyclic carbamate linker **235** or an amino-acid motive **236**.

The biological assays showed no correlation between the activity of these compounds on tubulin and their cytotoxicity, suggesting the existence of cellular targets which should be elucidated.

The steganacine and steganone are two natural lignin lactones isolated from *Steganotaenia araliacea*, which interact also with the colchicine binding site of tubulin, and therefore exert anti-mitotic activities. These compounds have a significant cytotoxicity in vivo against P388 leukemia in mice, and in vitro against several cancer cell lines, among them the human nasopharynx carcinoma [89].

**Scheme 50:**
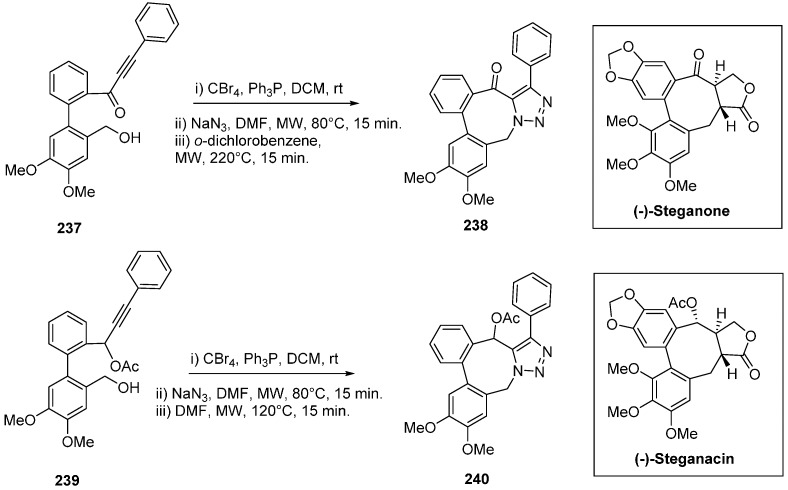
MW-assisted synthesis of Steganacin and 8-aza-Steganone.

Van der Eycken et al. [90] reported recently a MW-assisted nine-step protocol for the synthesis of unnatural derivatives from these two molecules **238**, **239** (Scheme 50). The intramolecular cyclization step was carried out following a three-step pseudo one-pot sequence. In the first step, the primary alcohol is converted into bromine through a conventional Appel reaction at room temperature, and using CBr_4_ and Ph_3_P. The resulting intermediate undergoes a MW-assisted nucleophilic substitution, using NaN_3_ in DMF at 80 °C. 

The last step, consists on a MW-assisted intramolecular Hüisgen 1,3-dipolar cycloaddition between the azide group and the acetylene. This reaction requires higher temperature and leads to the final compound with lower yield when using the ketone derivative **237** (43%) and not the acetate derivative **239** (76%). It is also noteworthy that under conventional heating this intramolecular cycloaddition doesn’t occur. In a further work, the same group [91] has explored MW-enhanced Suzuki–Miyaura cross-coupling and ring-closing metathesis (RCM) as key steps for the synthesis of *N*-shifted buflavine analogues. In this preparation, the rigid 8-membered cycle has been obtained through RCM. The best conversion rates have been obtained using 3 mol % Grubbs II catalyst and under MW-heating for 5 min (150 W); indeed, a conventional heating leads to the products with poor yields and requires several hours in refluxing toluene. 

**Scheme 51:**
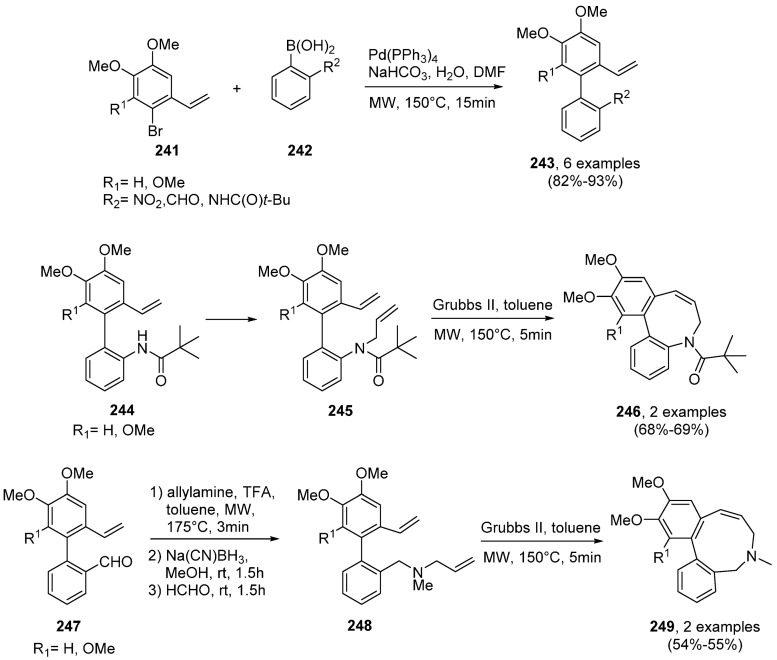
Synthesis of buflavine analogues through a Suzuki-Miyaura cross-coupling and MW-activated RCM sequence.

In 2007, this group reported also the synthesis of ring-expanded and *N*-shifted buflavine analogues (Scheme 51) [92]. This approach includes two key steps: (i) a Suzuki-Miyaura cross-coupling reaction between electron-rich aryl halides **241** and *ortho*-substituted boronic acids **242** affording the corresponding series of biaryl derivatives **243**, **244** and **247**; and (ii) a RCM reaction.

The buflavine derivatives encompassing a 8-membered cycle **246**, were obtained by allylation of biaryls **244** and subsequent RCM using Grubb’s II catalyst. The buflavine derivatives encompassing a 9-membered cycle **249**, were obtained through a reductive amination of the biaryl aldehydes **247** leading to the tertiary amines **248**, followed by the RCM reaction. The MW-activation of the RCM is highly beneficial to generate these highly strained ring systems, and leads to the targeted compounds with higher conversion rates compared to those obtained with a conventional heating.

**Scheme 52:**
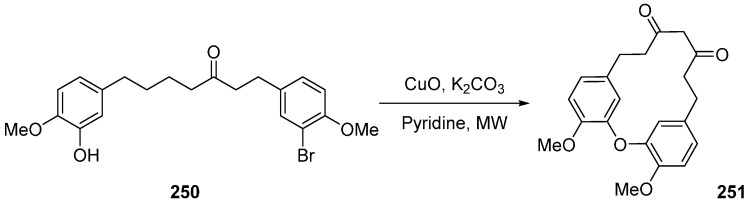
MW-assisted Ullmann coupling leading to macrocyclic diaryl ethers.

**Figure 12:**
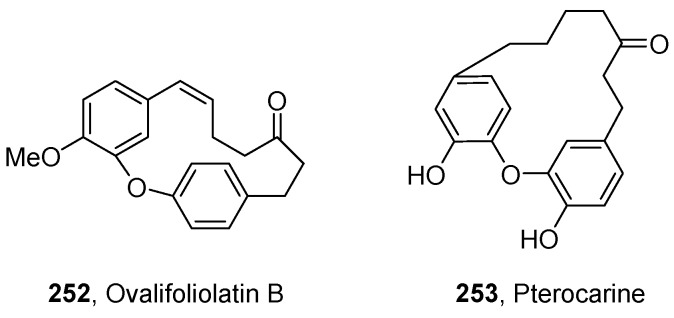
Relevant examples of macrocyclic diaryl ethers as natural compounds.

More recently, Shen et al. [93] have performed the synthesis of macrocyclic diaryl ethers **251** via intramolecular and/or bimolecular Ullmann coupling under MW-irradiation (Scheme 52). The authors reported higher yields and shorter reaction times compared with conventional heating and sealed tubes. These macrocyclic diaryl ethers are commonly found in natural compounds (Figure 12) and have been reported to mediate a broad range of biological activities [94,95,96].

#### 2.2.3. MW-Assisted Syntheses of Phthalocyanines

**Scheme 53:**
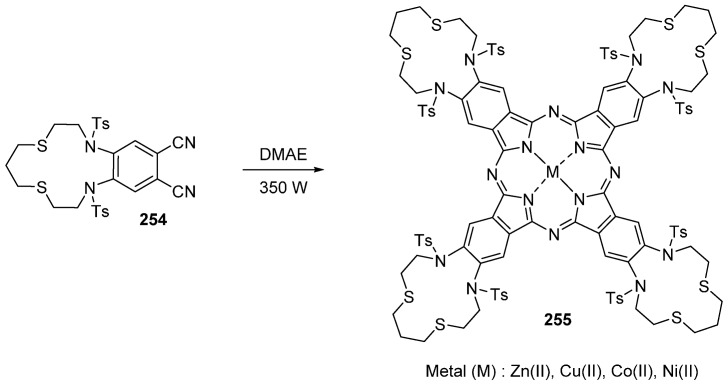
MW-assisted synthesis of metallophthalocyanine.

Another application of MW-irradiation for enhanced macrocycle syntheses has been investigated by Kantekin’s group, in the case of metal-free phthalocyanine syntheses [97,98,99,100]. Indeed, this novel series of tetrasubstituted metal-free phthalocyanines and metallophthalocyanines is obtained through the MW-activated cyclotetramerization of corresponding dicyano derivatives **254** in 2-(dimethylamino) ethanol for 10 min. The metallophthalo-cyanines **255** were obtained using corresponding anhydrous metal salts NiCl_2_ and ZnCl_2_, CoCl_2_, CuCl_2_, under MW-irradiation in 2-(dimethylamino)ethanol at 175 °C and 350 W, for 10 min (Scheme 53) [101].

**Scheme 54:**
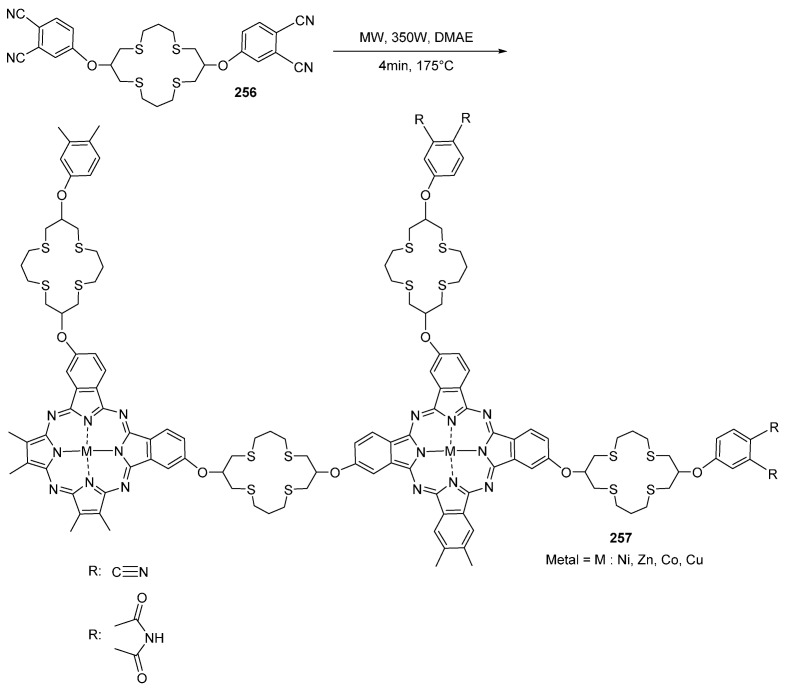
MW-assisted synthesis of metal-free phthalocyanine polymer.

The same group have reported the synthesis of the metal-free phthalocyanine polymer **257** by the reaction of the tetranitrile monomer **256** with 4-({11-[3-cyano-4-(cyanomethyl)phenoxy]-1,5,9,13-tetrathiacyclohexadecan-3-yl}oxy)phthalonitrile in 2-(dimethylamino)ethanol with a set of anhydrous metal salts in DMAE (Scheme 54) [102].

**Scheme 55:**
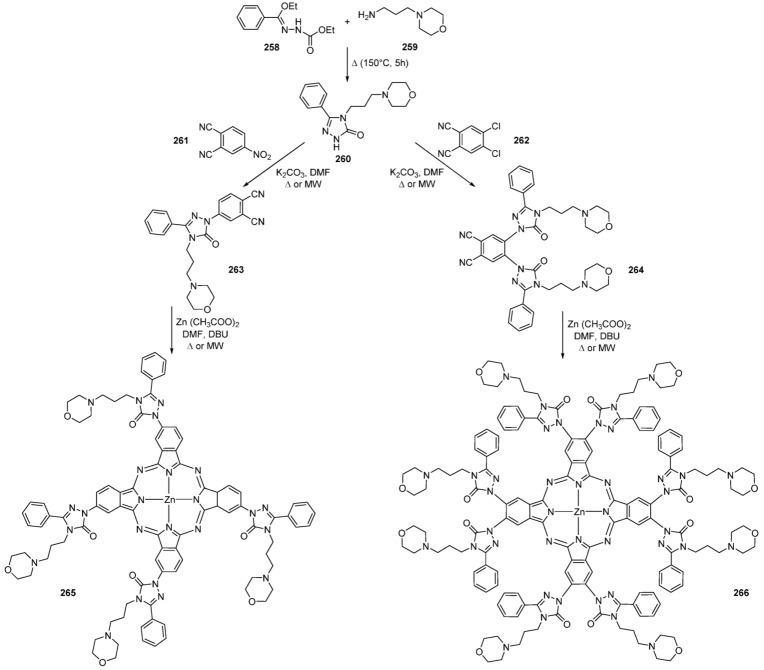
MW-assisted synthesis of phthalonitriles and zinc (II) phthalocyanines.

Recently, novel phthalonitriles and zinc (II) phthalocyanines containng morpholine and/or triazole as pharmacophores have been synthesized by Kantar et al. [103]. In the first step of this synthesis, the triazole **260** has been synthesized through the treatment of compound **258** with 3-morpholinopropylamine **259**. Then, the mono- and disubstituted phthalonitrile derivatives **263** and **264** have been obtained by reacting triazole **260**, 4-nitro-1,2-dicyanobenzene (**261**) or 4,5-dichloro-1,2-dicyanobenzene (**262**) respectively, through base-catalyzed nucleophilic aromatic substitution. Lastly, zinc (II) phthalocyanines **265**, **266** have been synthesized from the corresponding phthalonitrile derivatives mixed with zinc acetate (Scheme 55). By comparison with conventional heating, the authors reported that MW-assisted syntheses result in higher yields and shortened reaction times. All compounds were screened for anti-xanthine oxidase (anti-XO) activities. The compound **263** exhibited promising XO inhibition with IC_50_ value as 0.082 mM. Anti-xanthine oxidase activity has not been observed in the case of the phthalocyanines **265** and **266**; this latter result is probably due to their potency to form aggregates and their poor solubility.

#### 2.2.4. MW-Assisted Syntheses of Calix-Type Derivatives

**Scheme 56:**
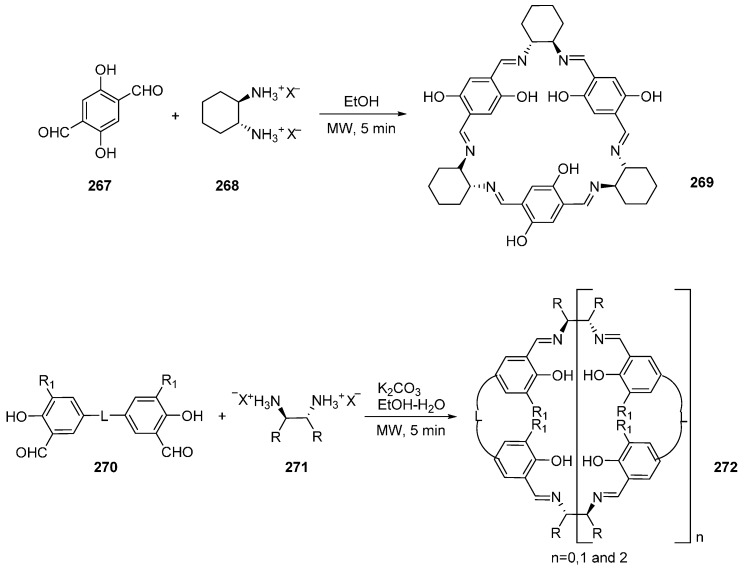
First example of a MW-activated calix-type synthesis.

The first example of a MW-activated calix-type synthesis has been reported by Srimurugan et al. [104]. In this pioneering work, macrocycle **269** and macrocycle **272** were obtained from dialdehydes **267**, **270** and chiral diamines **268**, **271**, and linked by a Schiff base (imine) as illustrated in Scheme 56.

**Scheme 57:**
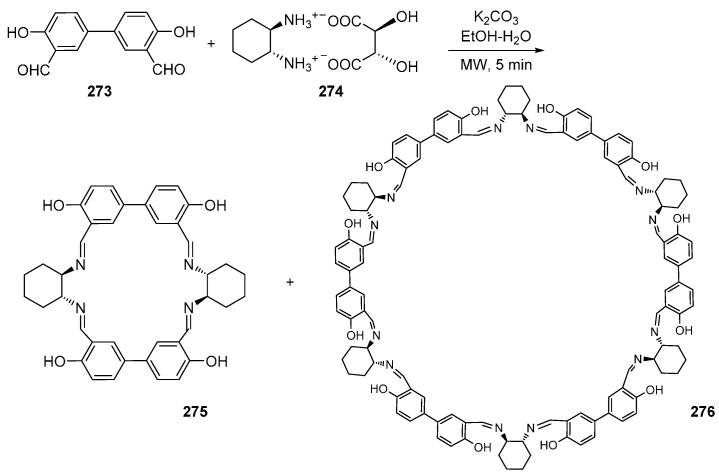
MW-assisted synthesis of 24- or new 72-membered chiral macrocyclic Schiff bases.

A similar approach has also been reported by the same group [105] for the synthesis of 24- or new 72-membered chiral macrocyclic Schiff bases through [2 + 2] or [6 + 6] cyclocondensations (Scheme 57). Practically, the MW-irradiation of a mixture of **273** and (1*R*,2*R*)-diammoniumcyclohexane mono-(+)-tartrate (**274**) in the presence of potassium carbonate afforded two main products **275** and **276** in respectively 14% and 36% isolated yield (Scheme 57). The corresponding thermal reaction produced the [2 + 2] macrocycle **275** as the major product, with only a few traces of the [6 + 6] macrocycle **276**.

The conventional syntheses of calixarenes occurs through a base-catalyzed condensation of substituted phenols with aldehydes. These reactions require high temperatures, in particular for the synthesis of a cyclic tetramers (calix[4]arene), usually considered as a thermodynamic cyclization product, conversely to the octameric product (calix[8]arene), which is a kinetic adduct.

**Scheme 58:**
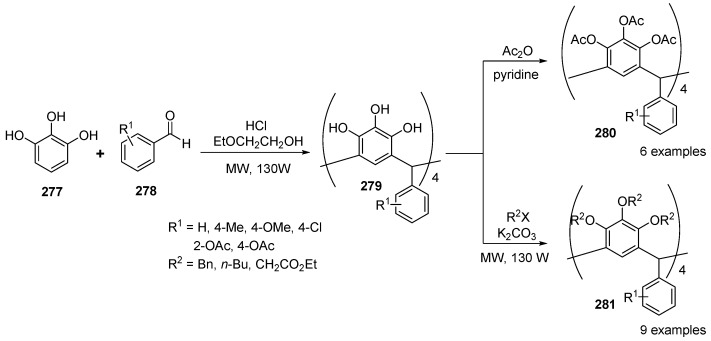
Green synthesis of calix[4]arenes.

In this context, some green procedures of variously substituted calix[4]arenes have been published in the last decade. A pioneering work, reported by Yan and co-workers in 2007 [106], described the preparation of pyrogallol[4]arenes **280**, **281**, which have been synthesized by reacting equal molar amounts of pyrogallol (**277**) and substituted aromatic aldehydes **278** in an acidic medium and under MW-irradiation (130 W) (Scheme 58). The expected calix[4]arenes **279** have been isolated in good yields (66%–89%). The reaction times are shortened to only 2–4 min, which is a major improvement by comparison with conventional heating. The subsequent conventional acylation or MW-assisted alkylation of the 12 hydroxy groups, afford compounds **280** and **281**, which are soluble in organic media and have been fully characterized by the authors.

**Scheme 59:**
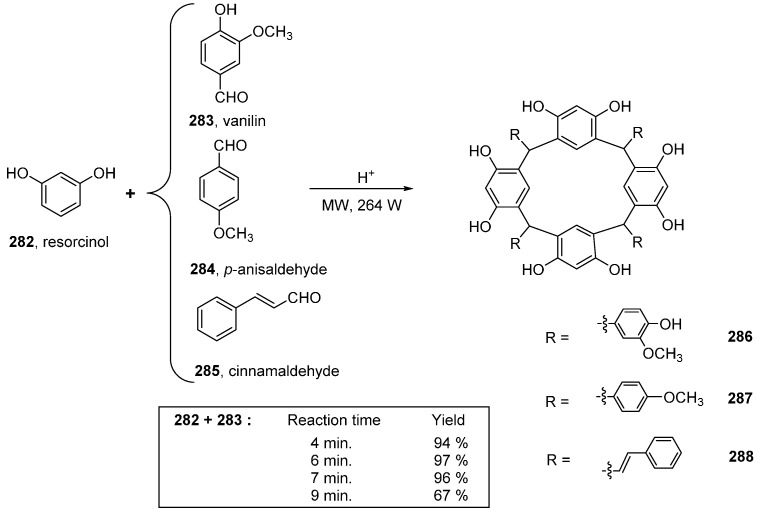
Green synthesis of calix[4]resorcinarenes.

It is noteworthy that similar approaches are still used, as illustrated by the recently published MW-assisted synthesis of three series of calix[4]resorcinarenes **286**–**288**. These derivatives have been isolated by mixing resorcinol (**282**) with different polysubstituted aromatic aldehydes (including vanillin (**283**), cinnamaldehyde (**285**) and *p*-anisaldehyde (**284**)) as illustrated in Scheme 59 [107]. Interestingly, the authors underlined in this study the relevance of the reaction time. Indeed, a longer MW-irradiation time leads to the significant reduction of the conversion rate.

Finally, it worth mention that MW-irradiation processes have been widely used to functionalize already formed calix[4]arenes (e.g., alkylation of hydroxyls [107,108,109,110] or click chemistry linkage [111]).

## 3. Conclusions

The advent of MW technology has profoundly changed the field of organic chemistry. The combination, on the one hand of the prompt and homogeneous heating of the reactants, and on the other hand of the MW non-thermal effects, has allowed the development of new series of highly functionalized molecules which could not by synthesized under conventional heating. In addition, the expected compounds are generally obtained in high yields and excellent purity after shortened reaction times. Moreover, most of the MW-assisted syntheses are highly regio- chemo- and stereo-selective. Importantly, MW-heating is also compatible with green chemistry procedures such as solid-phase and/or solvent-free syntheses. 

Macrocycles form a class of natural compounds and synthetic molecules with a large scope of potential therapeutic applications. Therefore, their syntheses have been extensively studied; nevertheless, the cyclisation step is generally very detrimental in terms of yield and purities. Indeed, these reactions are usually characterized by harsh conditions (long reaction times and elevated temperatures and prolonged heating), expensive reagents and solvents, poor regio-, chemo- and stereoselectivity leading to several side products and lastly a marked difficulty in recovering and re-using the catalysts.

**Figure 13:**
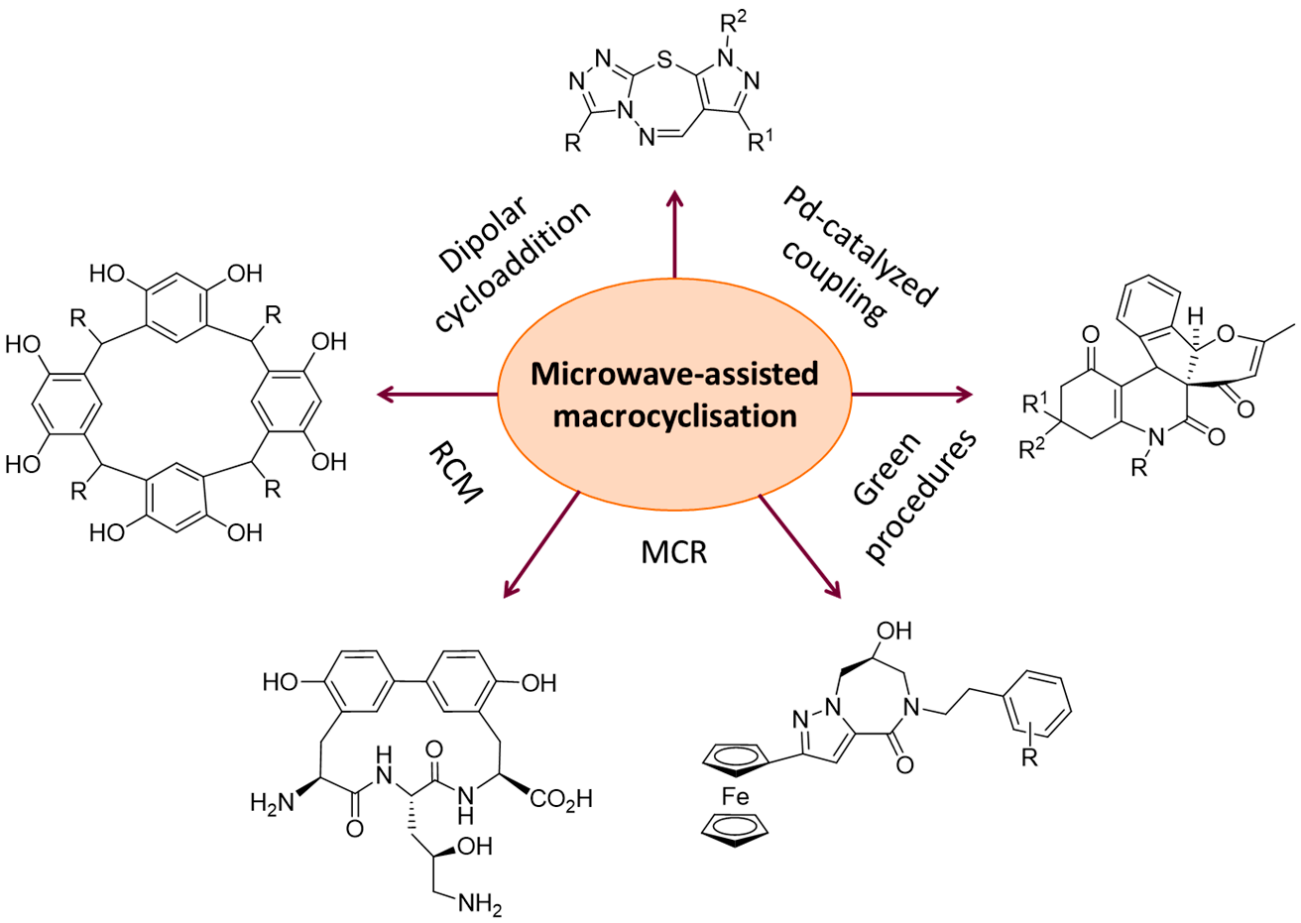
The large scope of the MW-assisted macrocyclisations.

By contrast, the use of MW-assisted procedures leads to dramatic optimizations for this specific macrocyclisation step (Figure 13). Taking into account the popularity of MW technology and the number of increasing articles devoted to this topic we selected for this review only relevant examples, in a non-exhaustive way, to illustrate the huge potential of this technology in modern heterocyclic chemistry. This will undoubtedly open the way to other applications and to the design and elaboration new scaffolds and to identify new bioactive molecules. 

## Abbreviations

The following abbreviations are used in this manuscript

Ac	Acetyle
Ala	Alanine
Alloc	Allyloxycarbonyl
Arg	Arginine
Asn	Asparagine
Asp	Aspartic acid
BET	Bacterial endotoxin test
BINAP	2,2′-Bis(diphenylphosphino)-1,1′-binaphthyl
Bn	Benzyl group
Boc	*tert*-Butyloxycarbonyl
Bu	Butyl
Cbz	Carboxybenzyl
CCK2	Cholecystokinin B
CNS	Central Nervous System
Cy	Cyclohexyl
Cys	Cysteine
Cu(Phen)(PPh_3_)Br	Bromo-(1,10-phenanthroline-*N*,*N*′)(triphenylphosphine)cuprate
dba	Dibenzylideneacetone
DBU	1,8-Diazabicyclo[5.4.0]undec-7-ene
DCB	1,4-Dichlorobenzene
DDA	Diketene acetone adduct
DCM	Dichloromethane
DIEA	*N*,*N*-Diisopropylethylamine
DIPCDI	*N*,*N*-Diisopropylcarbodiimide
DMAE	Dimethylethanolamine
DMA	Dimethylacetamide
DMF	*N*,*N*-Dimethylformamide
DMSO	Dimethyl sulfoxide
DNA	Deoxyribonucleic acid
Dppf	1,1′-Bis(diphenylphosphino)ferrocene
dr	Diastereomeric ratio
ee	Enantiomeric excess
eq.	Equivalent
Et	Ethyl
Fmoc	Fluorenylmethyloxycarbonyl
F-SPE	Fluorine solid phase extraction
EtOH	Ethanol
GII	Grubbs II catalyst
Gly	Glycine
HATU	1-[Bis(dimethylamino)methylene]-1*H*-1,2,3-triazolo[4,5-*b*]pyridinium-3-oxid hexafluorophosphate
HBS	Hydrogen-bond surrogate
HBTU	2-(1*H*-Benzotriazol-1-yl)-1,1,3,3-tetramethyluronium hexafluorophosphate
HCT 116	Human colon carcinoma cells
His	Histidine
HMBA	Hydroxymethylbenzoic acid
HGII	Hoveyda-Grubbs II catalyst
HOBt	Hydroxybenzotriazole
IC_50_	Half maximal inhibitory concentration
IL	Ionic Liquid
KSAc	Potassium thioacetate
Lys	Lysine
MBHA	4-Methylbenzhydryl amine
*m*-CPBA	*meta*-Chloroperoxybenzoic acid
MCR	Multi-component reaction
Me	Methyl
MeOH	Methanol
MIC	Minimum Inhibitory Concentration
Ms	Mesyl
mTOR	Mammalian Target of Rapamycin
min	Minute
MW	Microwave
NADH	Nicotinamide Adenine Dinucleotide Hydride
Nle	Norleucine
Pbf	2,2,4,6,7-Pentamethyldihydrobenzofuran-5-sulfonyl
Pd/C	Palladium on carbon
PEG	Polyethylene glycol
Ph	Phenyl
Phe	Phenylalanine
PheD	D-Phenylalanine
PMB	*p*-Methoxybenzyl
PPE	Polyphosphoric ester
PPh_3_	Triphenylphosphine
Pr	Propyl
PSSA	Polystyrene sulfonic acid
PTH1R	Parathyroid Hormone 1 Receptor
PyBOP	Benzotriazol-1-yl-oxytripyrrolidinophosphonium hexafluorophosphate
*p*-TsOH	*para*-Toluenesulfonic acid
RCM	Ring closure metathesis
r.t	Room temperature
Ser	Serine
SES	The 2-(trimethylsilyl)ethanesulfonyl group
S_N_Ar	Nucleophilic aromatic substitution
SPPS	Solid Phase Peptide Synthesis
TBAB	Tetrabutylammonium bromide
TFA	Trifluoroacetic acid
Thr	Threonine
TIPS	Triisopropylsilyl
TMS	Trimethylsilyl
Trp	Tryptophan
Trt	Trityl (triphenylmethyl)
Ts	Tosyl
THF	Tetrahydrofuran
XO	Xanthine Oxidase
